# “*Candidatus* Hydrogenisulfobacillus filiaventi” strain R50 gen. nov. sp. nov., a highly efficient producer of extracellular organic compounds from H_2_ and CO_2_

**DOI:** 10.3389/fmicb.2023.1151097

**Published:** 2023-03-24

**Authors:** Carmen Hogendoorn, Arjan Pol, Rob de Graaf, Paul B. White, Rob Mesman, Peter M. van Galen, Theo A. van Alen, Geert Cremers, Robert S. Jansen, Mike S. M. Jetten, Huub J. M. Op den Camp

**Affiliations:** ^1^Department of Microbiology, RIBES, Radboud University, Nijmegen, Netherlands; ^2^Department of Synthetic Organic Chemistry, IMM, Radboud University, Nijmegen, Netherlands; ^3^Department of Systems Chemistry, IMM, Faculty of Science, Radboud University, Nijmegen, Netherlands

**Keywords:** acidophilic, autotroph, hydrogenase, amino acids, volcanic soil, *Hydrogenisulfobacillus*

## Abstract

Production of organic molecules is largely depending on fossil fuels. A sustainable alternative would be the synthesis of these compounds from CO_2_ and a cheap energy source, such as H_2_, CH_4_, NH_3_, CO, sulfur compounds or iron(II). Volcanic and geothermal areas are rich in CO_2_ and reduced inorganic gasses and therefore habitats where novel chemolithoautotrophic microorganisms for the synthesis of organic compounds could be discovered. Here we describe “*Candidatus* Hydrogenisulfobacillus filiaventi” R50 gen. nov., sp. nov., a thermoacidophilic, autotrophic H_2_-oxidizing microorganism, that fixed CO_2_ and excreted no less than 0.54 mol organic carbon per mole fixed CO_2_. Extensive metabolomics and NMR analyses revealed that Val, Ala and Ile are the most dominant form of excreted organic carbon while the aromatic amino acids Tyr and Phe, and Glu and Lys were present at much lower concentrations. In addition to these proteinogenic amino acids, the excreted carbon consisted of homoserine lactone, homoserine and an unidentified amino acid. The biological role of the excretion remains uncertain. In the laboratory, we noticed the production under high growth rates (0.034 h^−1^, doubling time of 20 h) in combination with O_2_-limitation, which will most likely not occur in the natural habitat of this strain. Nevertheless, this large production of extracellular organic molecules from CO_2_ may open possibilities to use chemolithoautotrophic microorganisms for the sustainable production of important biomolecules.

## Introduction

Fossil fuels, such as coal, natural gas and oil, are used in energy generation, transportation and for the production of chemicals and this contributes to more greenhouse gas emissions, global warming and an imbalanced global carbon cycle (SEI, 2021). To reduce global warming, usage of fossil fuels should be replaced by more sustainable resources, such as solar and wind power. However, the production of chemicals still largely depends on fossil fuels. Alternatively, chemicals can be produced by fixing CO_2_ into organic compounds. Methods to fix CO_2_ are currently being explored and involve the chemical and biological fixation of CO_2_ into larger molecules (Appel et al., 2013; Hepburn et al., 2019; Ullah et al., 2019).

Plants and several prokaryotic microorganisms can fix CO_2_ for the production of organic compounds (biomass). Using plants for the production of chemicals will require massive amounts of land, ultimately resulting in competition between biomass production, agriculture and nature. Therefore, microorganisms, and more specifically photosynthetic microalgae, can be used for the production of starch, carotenoids, fatty acids, proteins, antioxidants and pigments (Mooij et al., 2013; Gangl et al., 2015). Fixing CO_2_ to produce larger biomolecules requires energy. Photoautotrophic microorganisms use light as energy source for this activation. Chemolithoautotrophic microorganisms, can use reduced inorganic compounds, such as H_2_, CH_4_, NH_3_, CO, sulfur compounds or iron(II) for activation of CO_2_ (Friedrich and Schwartz, 1993; Khadem et al., 2011).

Volcanic and geothermal terrestrial soils are hot, acidic and emit several gasses, including high amounts of CO_2_ and the reduced gasses H_2_, CH_4_, CO and H_2_S (D’Alessandro et al., 2009; Magro et al., 2013; Bergfield et al., 2014; Gagliano et al., 2016). Despite the harsh conditions, these soils are home to a unique microbial community that can use the geothermal CO_2_ for carbon fixation and the reduced gasses as energy source (Picone et al., 2020). Metagenomic studies of Yellowstone National Park hot springs have shown the presence of a microbial community, which is enriched in genes encoding for H_2_ oxidation and CO_2_ fixation (Lindsay et al., 2019). Metagenomics of the geothermal soils of Pantelleria Island identified a high variety of hydrogenase genes and H_2_ consumption has already been observed in the top layers of geothermal soils (Gagliano et al., 2016; Picone et al., 2020).

Oxidation of H_2_ is catalyzed by the enzyme hydrogenase, whereby H_2_ is converted into two protons and two electrons. These enzymes are classified as [NiFe]-, [FeFe]- or [Fe]-hydrogenases depending on the metal ion in the active site (Lubitz et al., 2014). These hydrogenases can be further categorized into eight groups and 38 subgroups based on amino acid-based phylogenetic analyzes, metal-binding motifs, predicted genetic organization and reported biochemical characteristics (Greening et al., 2016). Uptake hydrogenases are involved in the oxidation of H_2_, whereby the generated energy can be used to fix inorganic carbon.

So far, seven CO_2_ fixation pathways have been discovered (Gong et al., 2016; Sánchez-Andrea et al., 2020). Three of these are involved in CO_2_ fixation under mostly anaerobic conditions, including the Wood-Ljungdahl pathway, the dicarboxylate/4-hydroxybutyrate cycle and the reductive TCA cycle. The three more predominant aerobic CO_2_ fixation pathways are the 3-hydroxypropionate cycle, the 3-hydroxypropionate-4-hydroxybutyrate cycle and the Calvin-Benson-Bassham (CBB) cycle. The widespread CBB cycle can be found among plants, algae, photosynthetic prokaryotes and many chemolithoautotrophic microorganisms. The key enzyme of the CBB cycle is the ribulose-1,5- bisphosphate carboxylase (cbb) composed of a large and a small subunit. In this pathway, three molecules of CO_2_ are converted to glyceraldehyde 3-phosphate at the expense of nine ATP molecules and six NAD(P)H molecules, which makes it the highest energy-consuming pathway for CO_2_ fixation (Berg, 2011; Erb and Zarzycki, 2018).

The aim of the present study was to enrich and isolate new thermoacidophilic, autotrophic microorganisms that serve as primary producers for the establishment of a microbial community and potentially produce extracellular compounds. To this end, we used aerobic chemostat cultivation with H_2_ as limiting substrate and CO_2_ as carbon source. A pure culture was obtained by serial dilution to extinction and resulted in the isolation of a novel bacterium of the phylum Bacillota, which we tentatively named “*Candidatus* Hydrogenisulfobacillus filiaventi” strain R50. “*Ca*. H. filiaventi” R50 is a chemolithotrophic microorganism that grows on H_2_, CO_2_ and O_2_ and produces large amounts of extracellular organic compounds, mainly amino acids.

## Materials and methods

### Geological setting

Pantelleria Island is the largest volcanic satellite island of Sicily, characterized by phenomena related to hydrothermal activities as fumaroles and passive degassing from geothermal soils. The main active area is called Favara Grande, with soil temperatures up to 115°C at 5 cm of depth and soil pH values as low as 3. The geothermal field passively degasses CO_2_, CH_4_ and H_2_ in order of magnitude of percent per volume unit. Soil samples were taken in June 2017 at Favara Grande from two sites, FAV1 (FAV1, 36°50′80”N; 11°57′170″E) and FAV2 (FAV2, 36°50′77”N; 11°57′160″E) (Gagliano et al., 2016), using a core sampler (diameter 1.5 cm), divided into subsections of 5 cm and stored in sterile 50 ml tubes.

### Enrichment and isolation

Soil (top 10 cm composite sample of site FAV1) was mixed with sterile medium of pH 3, using a volume ratio of 1:1. The medium was composed of 0.5 mM MgCl_2_, 0.5 mM CaCl_2_, 1 mM Na_2_SO_4_, 2 mM K_2_SO_4_, 1 mM (NH_4_)_2_SO_2_, and 1 mM NaH_2_PO_4_. The final trace element concentrations were 1 μM for CoCl_2_^.^6H_2_O, NaMoO_4_, Na_2_SeO_3_, CeCl_3_, and ZnSO_4_, 5 μM for MnCl_2_ and FeSO_4_, and 10 μM for CuSO_4_ and NiCl_2_. The pH was set to 3.0 by adding 1 M H_2_SO_4_. 4 ml of soil slurry was added to a bioreactor.

The bioreactor of 500 ml had a working volume of 325 ml and was stirred using a 4 cm stirring bar at 500 rpm. The reactor was operated at 75°C, pH 3.3 and supplied with a mixture of 80% CO_2_(v/v), 10% CH_4_ (v/v), 10% H_2_ at a flow rate of 5 ml/min. The CH_4_ flow was switched off after 4 weeks when it came apparent that CH_4_ was not consumed by the microbial community. Air was supplied to the reactor to reach a dissolved oxygen concentration of 4% air saturation. Two days after inoculation, the air supply was controlled to maintain a dissolved O_2_ concentration between 0.2 and 5% air saturation. After 12 days, the reactor was operated as chemostat with a dilution rate of 0.018 h^−1^. After steady state was reached, the temperature was gradually increased to 85°C. After the temperature had reached 85°C, the microbial culture became inactive and the bioreactor was re-inoculated with effluent and the temperature of the chemostat was stepwise decreased to 65°C. This resulted in a shift in population, as could be seen from microscopy. Coccoid cells disappeared and rod-shaped microorganisms started to appear. These microorganisms were further enriched and isolated using serial dilutions to extinction using 9 consecutive rounds. In the first six rounds, the reactor medium was used for the serial dilutions. From round seven onward, the medium was supplemented with 0.05 mM Na_2_S_2_O_3_ as a source of sulfur. 10 ml of medium was added to a 120 ml bottle containing 10% H_2_ (v/v), 10% CO_2_ (v/v), 3% O_2_ (v/v), and 77% N_2_ (v/v) in the headspace. These bottles were incubated at 55°C and 200 rpm shaking.

### Batch cultivation

Growth experiments were performed in 120 ml flasks with 20 ml medium. The headspace contained 10% (v/v) H_2_, 5% (v/v) O_2_, 5% (v/v) CO_2_, and 80% (v/v) N_2_. Bottles were incubated at 55°C, unless stated otherwise, in a shaking incubator operating at 200 rpm. To test the growth on organic substrates, H_2_ was replaced by 25 mM of organic substrate (formate, acetate, butyrate, methanol, ethanol, propanol, butanol, glucose, galactose, fructose, maltose, and succinate) or yeast extract (1 g/l). To test nitrogen fixation, medium without ammonium was used. To test for growth on urea as nitrogen source, ammonium was replaced by 2 mM urea.

### Batch and continuous cultivation in a bioreactor

Cultivation was performed in a 500 ml bioreactor (Applikon, Delft, Netherlands) with a working volume of 350 ml and the medium described above. The temperature was maintained at 55°C using a Peltier element. The pH was set to 3.0, measured by a pH electrode and maintained at pH 3.0 ± 0.1 by adding 0.2 M NaOH. The dissolved oxygen (DO) concentration was measured using a DO electrode, but remained 0% since the reactor was operated at O_2_-limiting conditions, while supplied with 2 ml/min air. Temperature and pH were controlled using the My-Control process controller (Applikon, Delft, Netherlands). The reactor was stirred at 2,000 rpm using a stirrer with two Rushton impellers. The reactor was supplied with 10 ml/min CO_2_: Argon (5%: 95%, v/v) and 3 ml/min H_2_. The O_2_-limited continuous cultivation was operated at a dilution rate of 0.034 h^−1^.To determine to growth rate at different dissolved oxygen concentrations, the DO was set to 0.2, 0.5, 3.5, or 7.5% dissolved air saturation and the reactor was operated as batch.

### Gas analysis

H_2_ in the headspace of the bottles and the in- and outflow of the chemostat cultures was measured using a HP 5890 gas chromatograph (Agilent, Santa Clara, California) equipped with a Porapak Q column (1.8 m, ID 2 mm) and a thermal conductivity detector. For this analysis, 50–100 μl gas samples were injected. To determine the CO_2_ and O_2_ consumption, 25 μl gas was injected and measured on an Agilent series 6,890 GC–MS (Agilent, Santa Clara, California) and analyzed as described before (Ettwig et al., 2008).

### Optical density, dry weight, total organic carbon, and total nitrogen

The optical density was measured using a Cary 50 UV–VIS spectrophotometer at a wavelength of 600 nm (Agilent, Santa Clara, California). Dry weight was determined by filtering 25 ml of culture over a pre-weighted 0.45 μm filter, drying at 60°C under vacuum and weight measurement. The total organic carbon and total nitrogen in the supernatant and in cell suspensions was determined using a TOC-L analyzer (Shimadzu, Kyoto, Japan) as described before (Mohammadi et al., 2017).

### Untargeted metabolomics

Supernatant and blank culture medium were diluted five-fold in methanol:acetonitrile (1:1, v/v) and analyzed in triplicate on an Agilent 1,290 LC system coupled to a 6,546 Q-ToF high-resolution mass spectrometer (Agilent) as described (Jansen et al., 2020). In brief, 2 μl sample was injected onto a Diamond Hydride Type C column (Cogent) and subjected to a 0.4 ml/min gradient of water with 0.2% formic acid (A) in acetonitrile with 0.2% formic acid (B) (0–2 min: 85% B, 3–5 min: 80% B, 6–7 min: 75% B, 8–9 min: 70% B, 10–11 min: 50% B, 11–14: 20% B, 14–24: 5% B, followed by 10 min re-equilibration at 85% B). Detection was performed from m/z 50–1,200 in the positive ionization mode, using a continuously infused solution of purine, trifluoroacetic acid, and hexakis for reference mass calibration. The resulting data were converted to mzXML format using ProteoWizard software (Chambers et al., 2012) and the two sample groups were compared using XCMS online (Tautenhahn et al., 2012) using centWave feature detection (15 ppm, 10–60s peak width, SN threshold 6, integration method 2), and obiwarp retention time correction (profStep 0.5). The resulting peak table was filtered for features that were more abundant in the culture supernatant compared to blank medium (fold-change >5, value of *p* <0.05) and present at a high level (max intensity >10^6^). From the resulting list of 12 features, 5 features representing isotopes or in-source fragments were manually removed, whereas the presence of one feature was not reproducible in subsequent analyzes. To identify the remaining 6 features, their accurate masses were queried in the metabolite database METLIN (Guijas et al., 2018).

### NMR analysis

All spectra were recorded on a Bruker AVANCE III 500 MHz spectrometer equipped with a Prodigy BB probe. Samples were collected as either 10x or 50x freeze-dried concentrates and dissolved in D_2_O containing TMSP as reference. ^1^H spectra were acquired with composite-pulse presaturation water suppression, 32 scans and a relaxation of 3 s. Multiplicity-edited HSQC spectra were acquired with 1,024 t1 increments using 25% NUS (256 real FIDs) and 8 scans per increment. HMBC spectra were acquired with 1,024 t1 increments using 50% NUS (512 real FIDs), 32 scans per increment and a nJCH of 8 Hz. 1H-1H DQF-COSY spectra were acquired with 512 t1 increments using 25% NUS (128 real FIDs) and 16 scans per increment. ^1^H-^1^H presaturated TOCSY spectra were acquired with 1,024 t1 increments using 25% NUS (256 real FIDs), 32 scans per increment and 80 ms of spin-lock mix time.

### Quantification of amino acids

An amino acid standard mixture containing 17 different L-amino acids at 1.25 and 2.5 μmoles/mL was obtained from Sigma Aldrich (AAS18-5 ml) and used to prepare calibration curves in blank culture medium for the Q-ToF LC–MS system described above. These calibration curves were used to calculate the concentration of the different amino acids in the supernatant of the R50 reactor. To this end, reactor supernatant was diluted 30x in culture medium. Of this solution 100 μl was diluted with 900 μl acetonitrile:methanol:water (40:40:20).

### Membrane inlet mass spectroscopy

The kinetics of hydrogen consumption were determined by membrane inlet mass spectrometry MIMS (Hiden HPR-40, Hiden Analytical, Warrington, United Kingdom). Detection took place by pulsed ion counting (PIC). For highest sensitivity the emission current was set between 250 and 450 μA. As an inlet probe a 1/8 inch stainless steel (SS) tube was used. The inlet probe (tube 1/8 inch, internal diameter 2.8 mm) was perforated at the end over 2 cm. The holes were covered with a piece of Dow Corning Q7-4750 silicon tubing (1.96 outer diameter, 0.25 mm wall thickness; Freudenberg Medical, VWR International, Amsterdam, NL). To apply the silicon on the probe tube, isopropanol was used as a lubricant. The probe was connected to a water trap, consisting of a 10 cm diameter coil (1/4 inch stainless steel tube) that was cooled inside a Dewar flask with solid carbon dioxide. Connections were made by Swagelok Unions with Vespel (85%)/Graphite (15%) ferrules. After connecting the probe to the vacuum inlet, the MIMS system was operated for a few days to reach a low and stable background signal, after which the chamber experiments were started.

A 500 ml glass vessel with a water-jacket was used as incubation chamber. The stainless steel head plate of the chamber was clamped to the chamber with a Viton O-ring in between. The head plate had three ports with Omni-Lok inverted cone fittings for pieces of 1/16 inch capillary peek tubes (0.76 mm inner diameter). After filling the chamber completely with medium, gassing was done at 40 ml/min with a long stainless steel capillary tube (0.7 mm diameter) through one of the ports, while the central port was open, for at least 30 min with a mixture of Ar and CO_2_ (95/5%) gas, and stirring with a stirrer bar (4 cm long) at 1000 rpm. Another port was used for adding deoxygenated medium, after the flushing procedure, by means of a 20 ml syringe. In this way all gas introduced during the gassing procedure was removed from the chamber. This port for gassing was later used as a capillary outlet for the medium when additions were done *via* the central tube by means of gastight glass syringes (Hamilton) fitted with 3 inch long needles (0.74 mm diameter). For calibrations and calculations, known amounts of hydrogen gas-saturated water were administered to the incubation chamber from 50 ml serum bottles containing 10 ml of water.

For measuring oxygen concentration in the chamber, an optical oxygen probe (DP-PST3) was used (PreSens - Precision Sensing, Regensburg, Germany). The probe was inserted through a 4 mm port that was sealed with a Viton O-ring. The sensor was connected to a Fibox 4 trace meter (PreSens). To adjust the initial oxygen concentration, oxygenated medium was added. For higher concentrations, pure oxygen gas was introduced. In the latter case, when the desired concentration was reached, the remaining gas was pushed out with medium from the 20 ml syringe. During the chamber incubations, the oxygen concentrations were maintained constant by adding oxygen saturated medium. Cells used for these experiments (250 μl) were obtained from a H_2_-limited chemostat culture (5% AS; OD_600_ = 1.6).

### Cryo EM

Cells were harvested from the O_2_-limited bioreactor and kept at 55°C until freezing. For each sample, 2 μl cell suspension was mixed with 0.5 μl 10 nm Protein A gold solution (CMC, UMC Utrecht) on a glow discharged Quantifoil R2/2 grid (Cu, 200#, Quantifoil Gmbh) and plunge frozen in liquid ethane using a Vitrobot Mk4 (FEI/Thermo Fisher) with controlled humidity (100%) at blot-force 1 with a blot time of 2.5 s. Frozen grinds were imaged in low dose in a JEOL-JEM2100 operating at 200 kV using a Gatan high-tilt cryo-holder.

### DNA sequencing and genome assembly

For DNA isolation, 2 ml cell suspension (OD_600_ 0.5–1.0) from the continuous culture was harvested by centrifugation (2 min, 14,000 x g) and resuspended in 100 μl sterile MQ water. DNA was extracted with the PowerSoil DNA isolation kit or the DNeasy Blood and Tissue kit according to the manufacturer’s instructions (Qiagen Benelux B.V, Venlo, The Netherlands). The quality and quantity of the DNA were analyzed using the Qubit (Thermo Fisher, Waltham, Massachusetts) and the Agilent 2,100 Bioanalyzer (Thermo Fisher, Waltham, Massachusetts). The genome was reconstructed using a combination of short-read paired-end Illumina sequencing and long-read Nanopore sequencing. For Illumina library preparation, the Nextera XT kit (Illumina, San Diego, California) was used according to the manufacturer’s instructions. Enzymatic tagmentation was performed starting with 1 ng of DNA, followed by incorporation of the indexed adapters and amplification of the library. After purification of the amplified library using AMPure XP beads (Beckman Coulter, Indianapolis, Indiana), libraries were checked for quality and size distribution using the Agilent 2,100 Bioanalyzer and the High sensitivity DNA kit. Quantitation of the library was performed by Qubit using the Qubit dsDNA HS Assay Kit (Thermo Fisher Scientific, Waltham, Massachusetts). The libraries were pooled, denatured, and sequenced with the Illumina MiSeq sequence machine (San Diego, California). Paired end sequencing of 2 × 301 base pairs was performed using the MiSeq Reagent Kit v3 (Illumina, San Diego, California) according to the manufacturer’s protocol. For Nanopore library preparation, 1–1.5 μg of DNA was used. The input DNA was checked for high molecular DNA and absence of degradation by agarose (0.5%) gel electrophoresis. For Nanopore sequencing, the DNA Library construction was performed using the Ligation Sequencing Kit 1D (SQK-LSK108) in combination the Native barcoding Expansion Kit (EXP-NBD103 or EXP-NBD104) according to the manufacturer’s protocol (Oxford Nanopore Technologies, Oxford, United Kingdom). The libraries were loaded and sequenced on a Flow Cell (R9.4.1) and run on a MinION device (Oxford Nanopore Technologies, Oxford, United Kingdom), according to the manufacturer’s instructions. Base calling after sequencing was done using the guppy_basecaller in combination with guppy_barcoder (Oxford Nanopore Technologies, Limited Version 2.3.7). The genome was assembled from Nanopore reads using Canu (v1.8) (Koren et al., 2017). Assembled contigs were first polished with Racon (v1.3.1) (Vaser et al., 2017) followed by two iterations of Pilon (v1.23) polishing with Illumina reads (Walker et al., 2014). The genome was annotated using the MicroScope platform (Vallenet et al., 2013) and annotations were checked manually. The genome of this strain is available from GenBank under accession number GCA_902809825.

### RNA sequencing and RNA-seq analysis

For transcriptome analysis, triplicate samples of 10 ml cell suspension (OD_600_ = 0.5) from the continuous culture (55°C, pH 3.0 with 0.2% AS, 7.5% AS or O_2_-limiting conditions) were harvested by centrifugation and mRNA was isolated using the RiboPure™-Bacteria kit according to the manufacturer’s protocol (Thermo Fisher, Waltham, Massachusetts). The quality and quantity of the RNA were analyzed using the Qubit (Thermo Fisher, Waltham, Massachusetts) and the Agilent 2,100 Bioanalyzer (Thermo Fisher, Waltham, Massachusetts). The transcriptome libraries were constructed using the TruSeq® Stranded mRNA Library Prep protocol (Illumina, San Diego, California) according to the manufacturer’s instructions. Total mRNA was used for library preparation and obtained libraries were checked qualitatively and quantitatively as described above. Pooled libraries were sequenced using the Illumina MiSeq sequence machine (Illumina, San Diego, California United States). For sequencing, the 151 bp sequence chemistry was performed using the MiSeq Reagent Kit v3 (Illumina, San Diego, California) according to the manufacturer’s protocol in one direction. CLCBio software (version 10.1.1, Qiagen, Aarhus, Denmark) was used to perform RNA-seq analysis. Gene expression levels were compared by calculating the reads per kilobase per million reads (RPKM) values for the CDSs and calculating the log2-fold to median (Mundinger et al., 2019). The raw reads are available *via* project number PRJNA616204.

### 16S rRNA gene analysis

The 16S rRNA gene of bacteria was PCR amplified from isolated DNA using the primers 616F (5’-AGAGTTTGATYMTGGCTCAG-3′) and 1492R (5’-GGTTACCTTGTTACGACTT-3′) using the PCR program; 5 min 94°C, 30 cycles 40 s at 96°C, 40 s 55°C, 40 s 72°C and finally 10 min 72°C. The 16S rRNA gene of the Archaea was PCR amplified from isolated DNA using the primers Arch21F (5’-TTCCGGTTGATCCYGCCGG-3′) and Arch915R (5’-GTGCTCCCCCGCCAATTCCT-3′) using the PCR program; 5 min 94°C, 30 cycles 40 s at 96°C, 40 s 54°C, 900 s 72°C and finally 10 min 72°C. The amplicons were cloned into the pGEM-T Easy cloning vector (Promega) and transformed into competent *E. coli* cells. After growth of the cells, the 16S rRNA amplicon in the vector was PCR amplified, cleaned (GeneJET PCR purification kit, Thermo Fisher, Waltham, United States) and sequenced using the Sanger sequencing platform (BaseClear B.V., Leiden, The Netherlands).

## Results

### Enrichment and isolation

**Figure 1:**
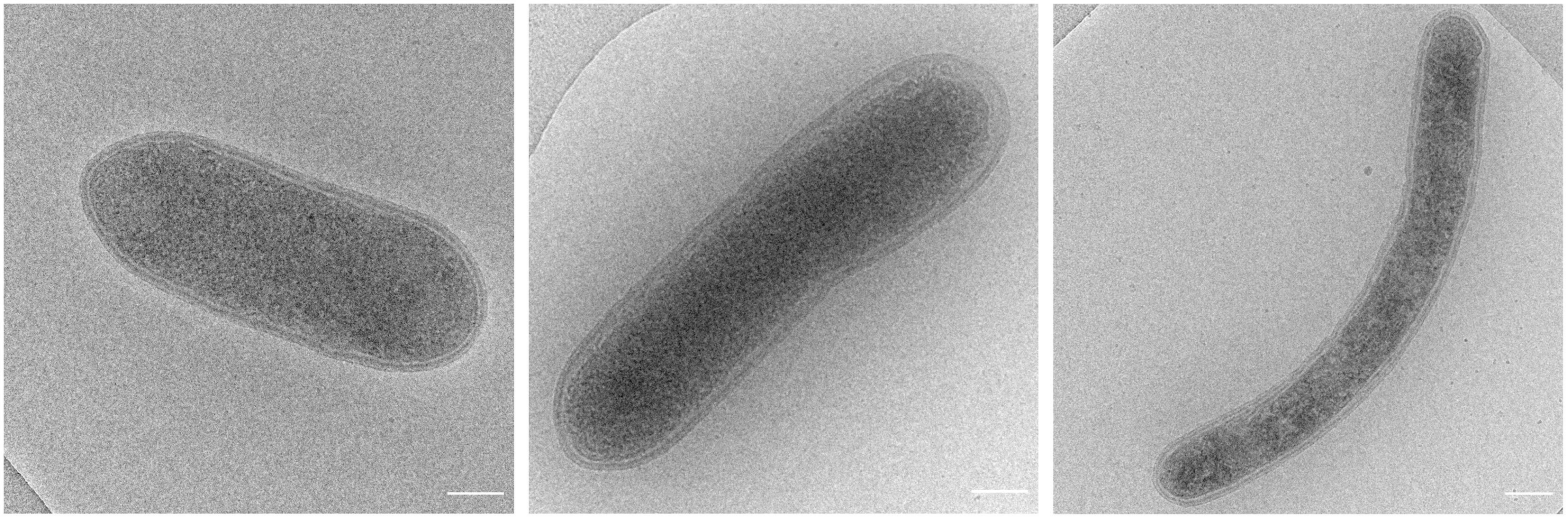
Cryo electron microscopy images of strain R50 (scale bar, 250 nm).

Geochemical analyzes and a metagenomic survey indicated the presence of high amounts of geothermal H_2_ and many genes encoding hydrogenases (Gagliano et al., 2016; Picone et al., 2020) in volcanic soils of Pantelleria Island. Therefore, we set out to enrich and isolate novel aerobic H_2_-oxidizing microorganisms using a continuous bioreactor with H_2_ as limiting substrate. As inoculum, soil from the geothermal active area Favara Grande on the island of Pantelleria, Italy, was used. The primary growth temperature was 75°C. Under these growth conditions, microscopic analysis showed that this microbial culture was dominated by a coccoid-shaped microorganism (Supplementary Figure S1). Analysis of the microbial culture indicated that the 16S rRNA gene of this microorganism had a high identity with *Acidianus* sp. (97–99%) (Supplementary Figure S2). The enriched *Acidianus* sp. could be isolated in pure culture by serial dilution but grew poorly in bottles despite additions of sulfide, cysteine, thiosulfate, sulfur, or yeast extract. The temperature optimum of 70–75°C of the *Acidianus* sp. dominated enrichment culture was determined by stepwise increasing the temperature with 5°C steps and measuring the maximum growth rate. However, after the temperature had reached 85°C, the culture became inactive and the bioreactor was re-inoculated using the collected effluent. Then, the temperature was stepwise decreased, but after the temperature had reached 65°C, the microbial culture changed. The coccoid-shaped *Acidianus* sp. disappeared and now rod-shaped bacteria started to dominate the culture (Figure 1). Some cells were more elongated. These bacteria were isolated by means of serial dilution to extinction. Serial dilutions up to six rounds did not result in a pure culture. Only after adding thiosulfate as a sulfur source, three more serial dilutions series resulted in the isolation of strain R50.

### Phylogeny

The genome of strain R50 was sequenced and assembled resulting in a closed genome of 2.6 Mb. The general features of this genome are compiled in Table 1. The four complete rRNA operons were highly similar. A total of 22 different tRNAs were identified, with 1 to 5 copies per tRNA type.

**Table 1:** Genomic features of Strain R50.

Feature	Value
Genome size (bp)	2,570,337
DNA coding (bp)	2,273,977
DNA G + C (%)	70.55
Total genes	3,013
Protein coding genes	2,756
rRNA genes (4 operons)	12
tRNA genes	55
Genes assigned to COGs	15

**Figure 2:**
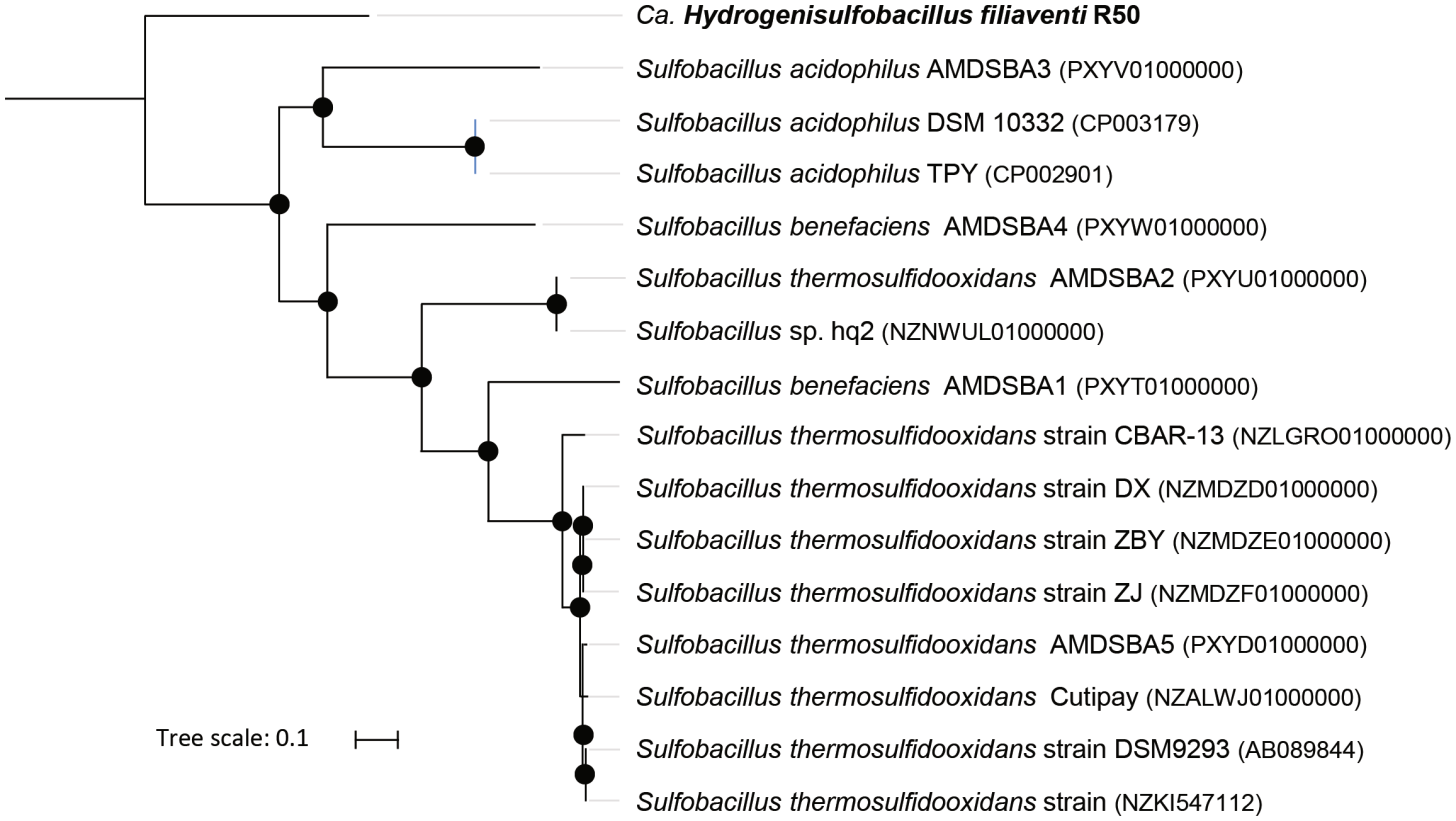
Up-to-date Bacterial Core Gene (UBCG) phylogenetic tree of strain R50 and members of the genus *Sulfobacillus*. *Hyphomicrobium denitrificans* was used to root the tree, but removed from the tree for clarity. The tree was constructed using RAxML. Bootstrap analysis was carried using 100 replications and percentage bootstrap values >95% are indicated by a black dot at the nodes.

**Figure 3:**
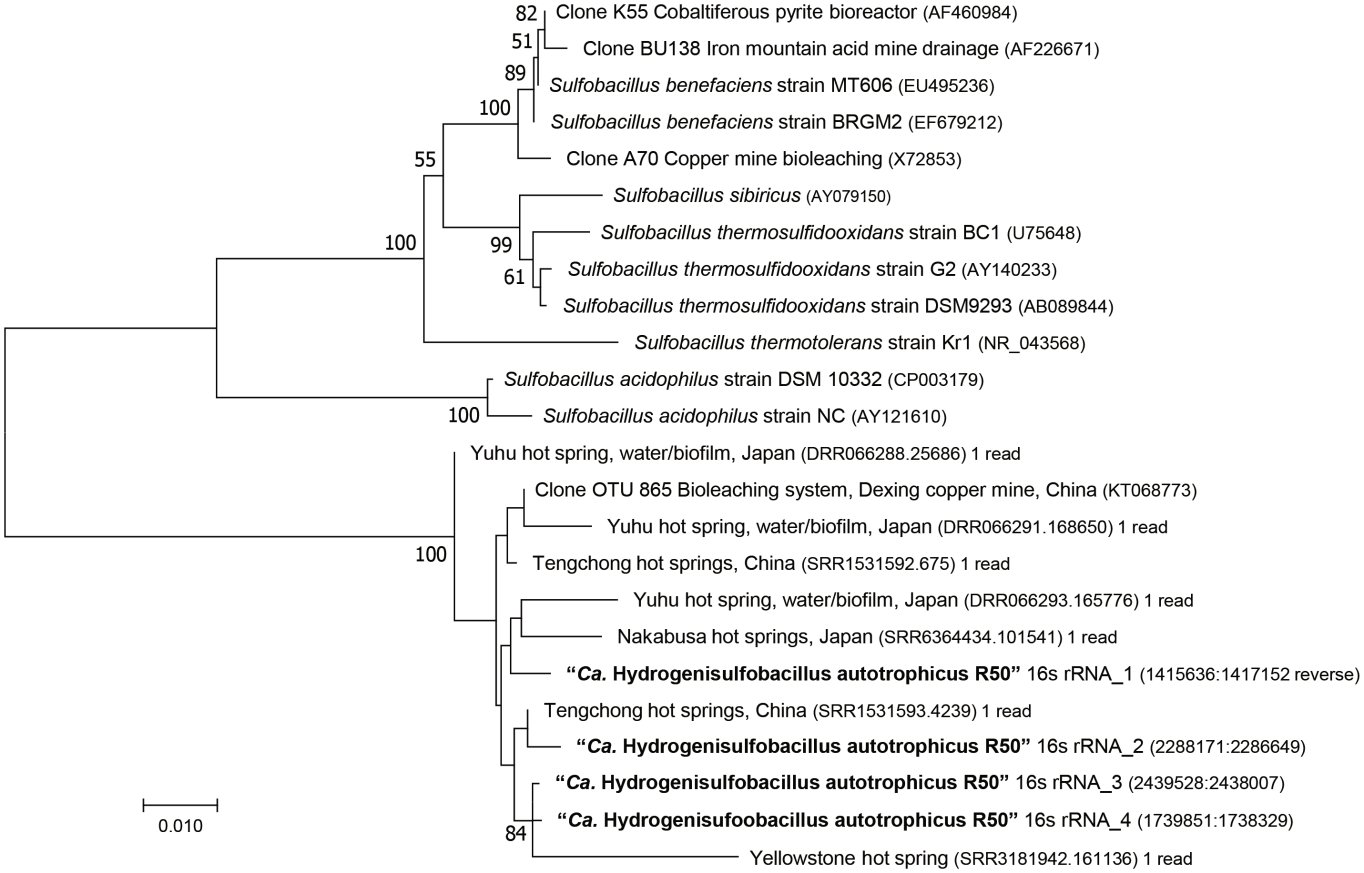
16S rRNA gene-based phylogenetic tree of Strain R50, representative *Sulfobacillus* species and environmental sequences. The evolutionary history was inferred using the Neighbor-Joining method. Bootstrap values above 50% (500 replicates) are shown next to the branches. Evolutionary distances were computed using the Jukes-Cantor method and the analysis was performed in MEGA7 (Kumar et al., 2016).

According to the GTDB classification (Chaumeil et al., 2019), strain R50 belongs to the phylum of *Bacillota* and to a new family [provisional R501 family (https://gtdb.ecogenomic.org/genome?gid=GCA_902809825.1)], but no genus or species could be attributed to this strain. Phylogenomic analysis of this strain shows that it falls outside of the genus *Sulfobacillus* (Figure 2). This is also supported by the Type (strain) Genome Server at DSMZ (Meier-Kolthoff and Göker, 2019; Supplementary Figure S3). The closest *Sulfobacillus* relative, *Sulfobacillus acidophilus* DSM 10332, shows 87% identity to the 16S rRNA gene of strain R50. No genomes or metagenome assembled genomes (MAGs) were found that are phylogenetically closer to strain R50 than members of the genus *Sulfobacillus*. To investigate if close relatives of this isolate can be found in other environments, the 16S rRNA gene (4 copies in the genome) was compared to prokaryotic 16S rRNA gene amplicon datasets retrieved using the integrated microbial NGS (IMNGS; Lagkouvardos et al., 2016) platform. The retrieved 16S rRNA amplicon sequences that showed a high identity with the 16S rRNA gene of strain R50 could be found in metagenomes of acid copper mines and hot springs (Figure 3). Based on the extended phylogenetic analysis, our isolate is a representative of a novel genus within the *Bacillota* for which we propose the name “*Candidatus* Hydrogenisulfobacillus filiaventi” R50 gen. nov., sp. nov.

### Description of “*Ca*. *Hydrogenisulfobacillus*” gen. nov.

“*Ca. Hydrogenisulfobacillus*” (*Hydrogenisulfobacillus*. N.L. n. *Hydrogeni*, Hydrogen; N.L. n. *bacillus* a small staff or rod; *Hydrogenisulfobacillus*, Hydrogen using microbe that is rod shaped and related to *Sulfobacillus* sp.).

### Description of “*Ca*. *Hydrogenisulfobacillus filiaventi*” sp. nov.

“*Ca.* Hydrogenisulfobacillus filiaventi” [fi.li.a.ven.ti. N.L. n. *filia*, daughter (from L. *filiae*, daughter); N.L. n. *venti*, wind (from L. *ventum*, wind); N.L. n *filiaventi*, daughter of the wind. “*Ca*. Hydrogenisulfobacillus filiaventi] R50 is isolated from the geothermal soils of the island of Pantelleria. The name of the island originates from Arab name Bint al-Riyãh, which means “daughter of the winds.”

### Physiology

The optimal temperature and pH of “*Ca*. H. filiaventi” R50 were determined during batch experiments with H_2_ as electron donor and CO_2_ as carbon source. The strain had an optimal growth temperature of 55°C (Supplementary Figure S4A) and grew only in a narrow pH range, namely between pH 2.5 and 4.0, with an optimum pH of 3.0 (Supplementary Figure S4B). No growth at pH 3 on any of the following organic substrates was observed; formate, acetate, butyrate, methanol, ethanol, propanol, butanol, glucose, galactose, fructose, maltose, succinate, and yeast extract. It was tested whether amino acids could serve as energy and nitrogen source, but no growth was observed on the amino acids such as alanine, arginine, aspartic acid, cysteine, glycine, glutamic acid, leucine, lysine, methionine, phenylalanine, proline, threonine, tyrosine, or valine. Only NH_4_^+^ could be used as nitrogen source and no growth was observed using nitrite, nitrate, urea or N_2_ as N-source. As S-source, sulfide, thiosulfate, tetrathionate, and cysteine could be used, but not sulfate. Cysteine could only be used as sulfur source, not as energy source. Spore formation was not observed.

To determine the stoichiometry of “*Ca*. H. filiaventi” R50, an O_2_-limited chemostat (D = 0.043 h^−1^) was started. Based on dry weight, 1.4 g DW was produced per mole H_2_ consumed. Under these conditions, 0.12 mol CO_2_ per mol H_2_ was fixed. Analysis of the total organic carbon showed that 0.54 mole organic carbon was excreted per mole CO_2_ consumed. This means that only 0.46 moles of carbon were assimilated into cellular biomass per mole CO_2_ consumed. Besides organic carbon, the total nitrogen could be determined. The N: C ratio of these organic compounds was 0.4. This narrows down the search of organic compounds that consists of carbon, nitrogen and most likely oxygen and hydrogen.

### Excretion of organic compounds

To identify the dominant organic metabolites excreted into the supernatant, we performed LC–MS untargeted metabolomics on culture supernatant and blank culture medium. After filtering for abundance and presence in culture supernatant, the six most abundant features were selected for further identification. Using accurate mass searches in the metabolite database METLIN, we found that five of these features had masses matching amino acids (Phe, Tyr, Ile/Leu, Val, Hse/Thr), while one feature matched the mass of an amino acid derivative (Hse-lactone).

**Figure 4:**
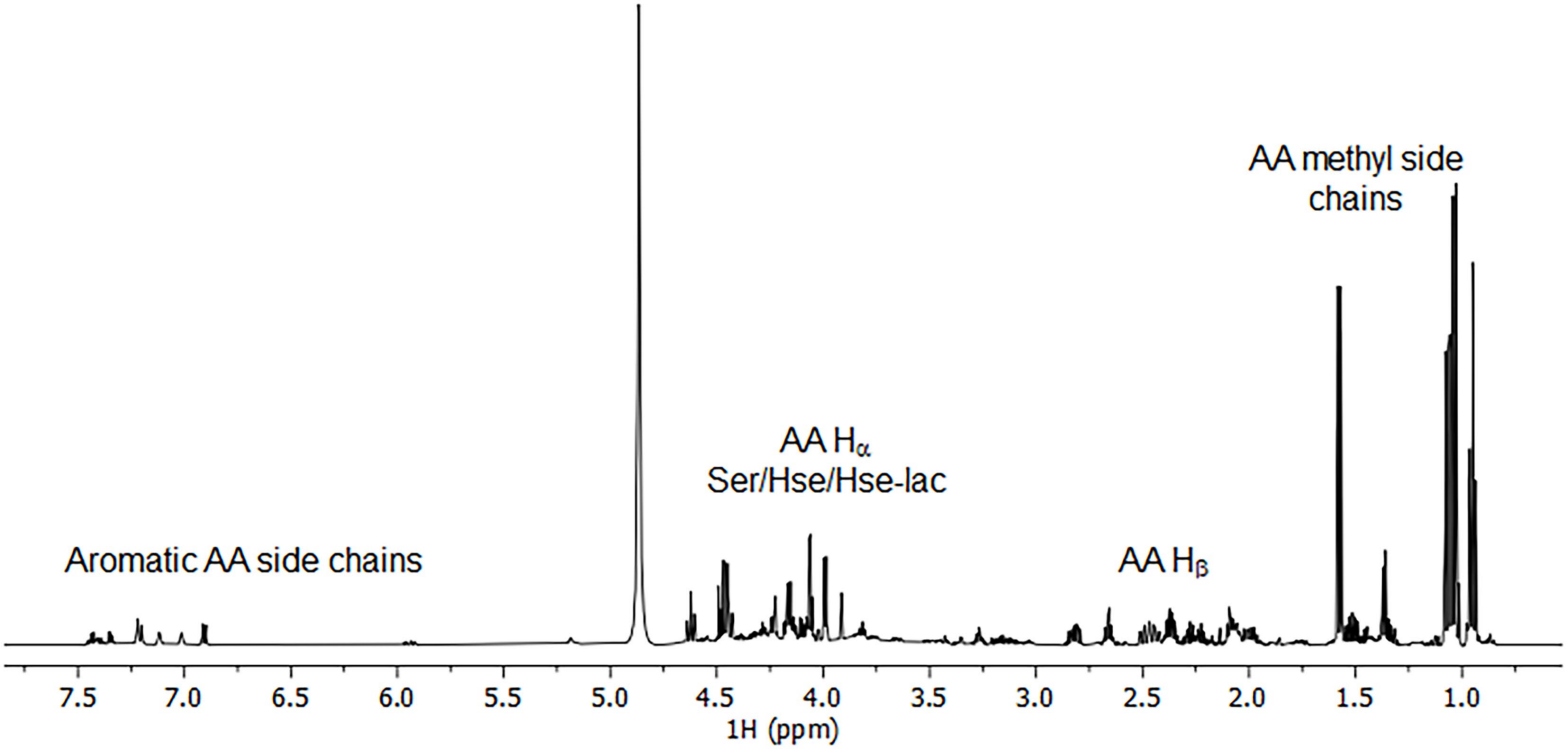
^1^H spectrum of 50x concentrated bioreactor supernatant.

**Figure 5:**
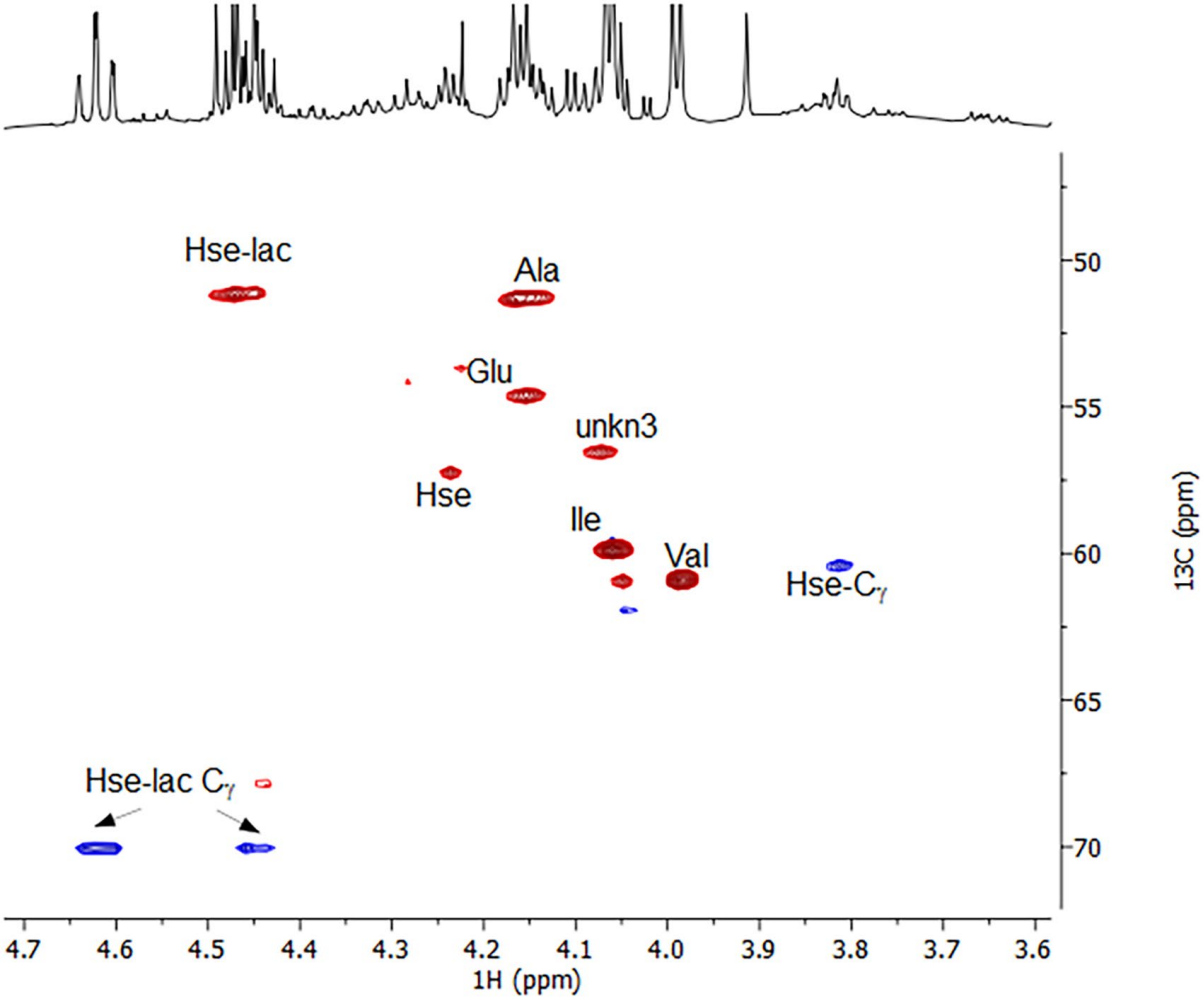
^1^H-^13^C multiplicity-edited HSQC spectrum highlighting the Hα-Cα correlations for the amino acids and the Hγ-Cγ correlations for Hse and Hse-lactone. Red corresponds to CH/CH_3_s and Blue to CH_2_s.

To corroborate these findings and extent the spectrum of excreted organic compounds, ^1^H as well as ^1^H-^1^H and ^1^H-^13^C correlation 2D NMR spectra were recorded from the culture supernatant (Figures 4, 5). From the 1D ^1^H spectrum, methyl-containing amino acids such as Val, Ala and Ile were immediately obvious. Similarly clear was the observation of the aromatic amino acids Tyr and Phe, albeit in much lower concentrations. Additionally, Lys and Glu/Gln were identified through their COSY and TOCSY ^1^H-^1^H correlations. While Lys was barely visible, Glu/Gln was relatively abundant. The assignment of Glu/Gln was complicated by the fact that they have very similar ^1^H and ^13^C chemical shifts but based on the LC–MS data, however, we assign the signal to Glu. Surprisingly, a large portion of the spectra could be assigned to the above proteinogenic amino acids. However, there were several sets of large resonances (Unk1-3) that appeared to have the structure of an amino acid but did not match the 20 proteinogenic amino acids. For Unk1-2, the large downfield chemical shifts for the γ ^1^Hs and ^13^Cs (Unk1: 3.85 ppm/60.4 ppm, Unk2: 4.4–4.6 ppm/70 ppm) suggested a structure with a directly attached O-atom, similar to Ser. Indeed, when comparing these sets of resonances to literature references for serine analogs and our LC–MS data, it could be concluded that Unk1 and Unk2 were homoserine (Hse) and homoserine lactone (Hse-lactone), respectively. This confirmed our LC–MS untargeted metabolomics results. The presence of Hse-lactone was intriguing because one would expect it to be unstable in the highly acidic growth medium, yet it was quite abundant. The hydrolysis product of Hse-lactone would be Hse, which could explain its presence. Together, Hse-lactone and Hse represent a major part of the excreted material. Another substantial species present was Unk3. HSQC and HMBC data revealed an amino acid backbone, however the side chain was revealed *via* COSY and TOCSY to be a simple ethyl group like homo-alanine (hAla) (Inada et al., 2019). Its presence could not be detected by LC–MS, so this metabolite remains unknown. Together, the LC–MS and NMR results thus provide a detailed overview, which shows that amino acids and their derivatives are the major excreted organic compounds.

To compare the identified excreted compounds with the total organic carbon, we quantified the excreted compounds using LC–MS (Table 2). Lysine, homoserine, homoserine lactone and unknown 3 were not quantified by LC–MS, but by comparison of NMR signals. Taken together, the amino acids detected by LC–MS and NMR represented 6.58 mM total carbon, which corresponds to 120% of the total excreted carbon determined by total organic carbon analysis.

**Table 2:** Quantification of excreted compounds in culture supernatant.

Compound	Concentration (μM)
Ala	264^a^
Glu	15.0^a^
Ile	327^a^
Lys	23.4^b^
Phe	36.0^a^
Tyr	39.6^a^
Val	239^a^
Hse	93.3^b^
Hse-lactone	222^b^
Unknown 3	119^b^

a Determined by LC–MS, ^b^Determined by NMR.

### Hydrogenases

**Figure 6:**
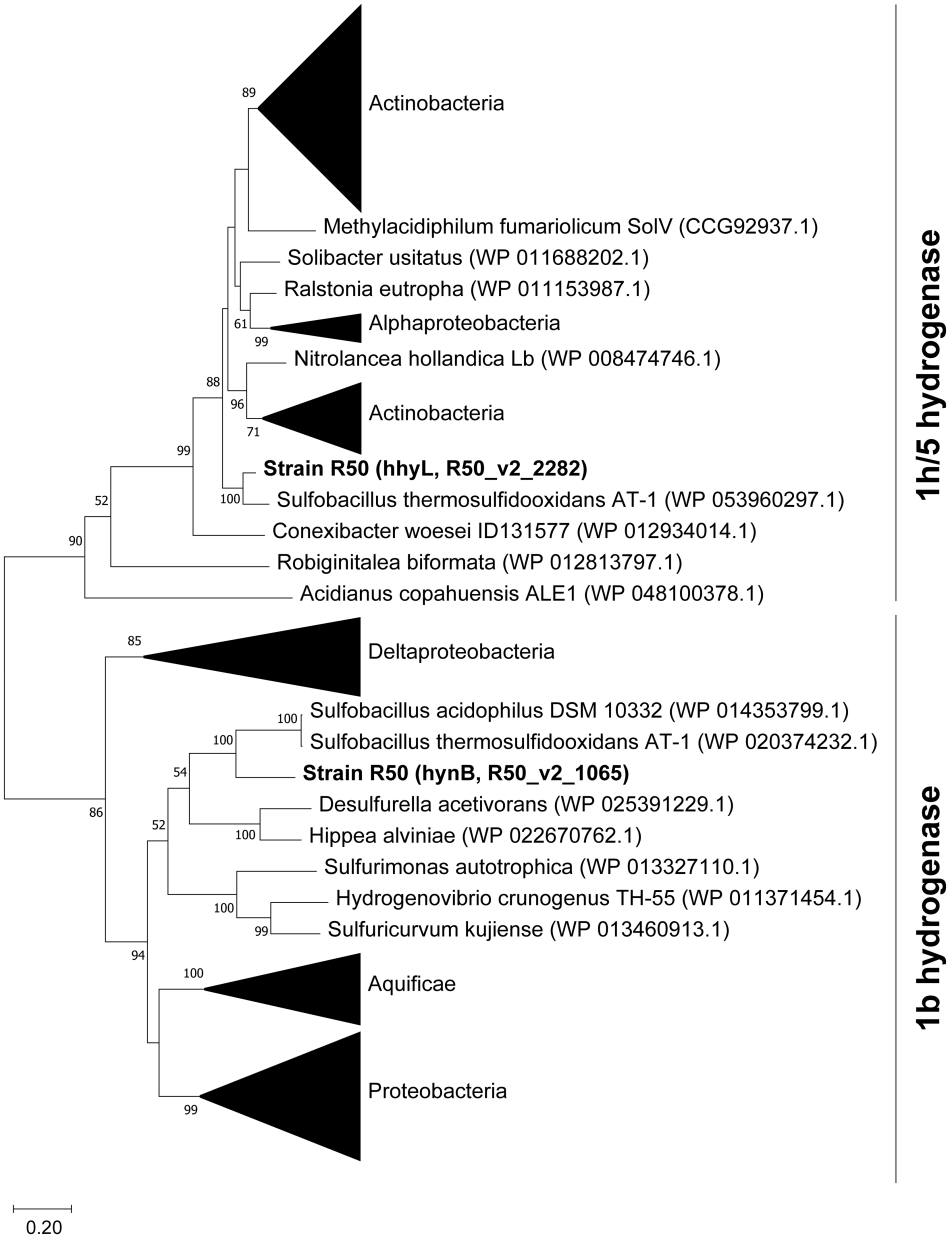
Phylogenetic analysis of the large subunits from group 1b and 1 h/5 hydrogenases. The tree was constructed using the Maximum Likelihood method (Saitou and Nei, 1987). Bootstrap percentage values above 50% (1,000 replicates) are given at each node. Evolutionary distances were calculated using the Poisson correction method (Zuckerkandl and Pauling, 1965). The analysis was performed using MEGAX (Kumar et al., 2018). The amino acid sequence of the [Fe]-hydrogenase of *Methanothermococcus thermolithotrophicus* (WP_018153721.1) was used to root the tree, but the branch was removed for clarification.

The assembled genome was analyzed for hydrogenase genes. Genes encoding two [NiFe]-hydrogenases were found, one belonging to group 1b (*hynA* and *hynB*) and one belonging to group 1 h/5 hydrogenases (*hhyS* and *hhyL*) (Figure 6). Phylogenetic analysis revealed that these [NiFe]-hydrogenases cluster with hydrogenases of Bacillota, with *Sulfobacillus* species as closest relatives (66–89% identity). The hydrogenases are closely related (~50% identity) to the characterized Campylobacterota hydrogenases of *Wolinella succinogenes*, *Helicobacter pylori*, *Campylobacter,* and *Sulfurospirillum multivorans*. The large subunit of the 1b hydrogenase, hynB, contains the active site motifs L1 (QRxCGVTxxH) and L2 (DPCxGCxVH). This L1 motif is typical for group 1b hydrogenases; however, the L2 motif contains a glycine where often an alanine is found (Greening et al., 2016). The large subunit of the 1 h/5 hydrogenase, *hhyL*, contains the L1 motif (TSRICGICGDNH) and L2 motif (SFDPCLPCGVH), which are conserved in group 1 h/5 hydrogenases (Greening et al., 2016). The group 1b hydrogenase operon shows besides the two catalytic subunits’ genes encoding HynC (cytochrome b subunit), HupD, HypC, a 4Fe-4S ferredoxin iron–sulfur-binding domain-containing protein, a conserved protein of unknown function, and HypA. Upstream of the operon separate HypC, HypE, and a TatA proteins are encoded. The cytochrome b subunit (HynC) shows five transmembrane helices. The group 1 h hydrogenase operon starts with the two catalytic subunits followed by genes encoding a small unknown protein, HypC, HypD, HypE, HypA, HYpB, a Rieske domain-containing protein, and eight additional conserved proteins of unknown function. All genes in both operons are constitutively expressed (Supplementary Table S1). The small subunits of the 1b- and 1 h-type hydrogenases show C-terminal motifs for FeS cluster binding, H-x(2)-C-x(19)-C-x(5)-C-x(19)-C-P-x(5)-C-x(2)-C and H-x(2)-C-x(24)-C-x(5)-C-x(8)-C-P-x(17)-C-x(2)-C, respectively. The 1b-type small subunit does not have a tat signal at the N-terminus pointing to a cytoplasmic location.

**Figure 7:**
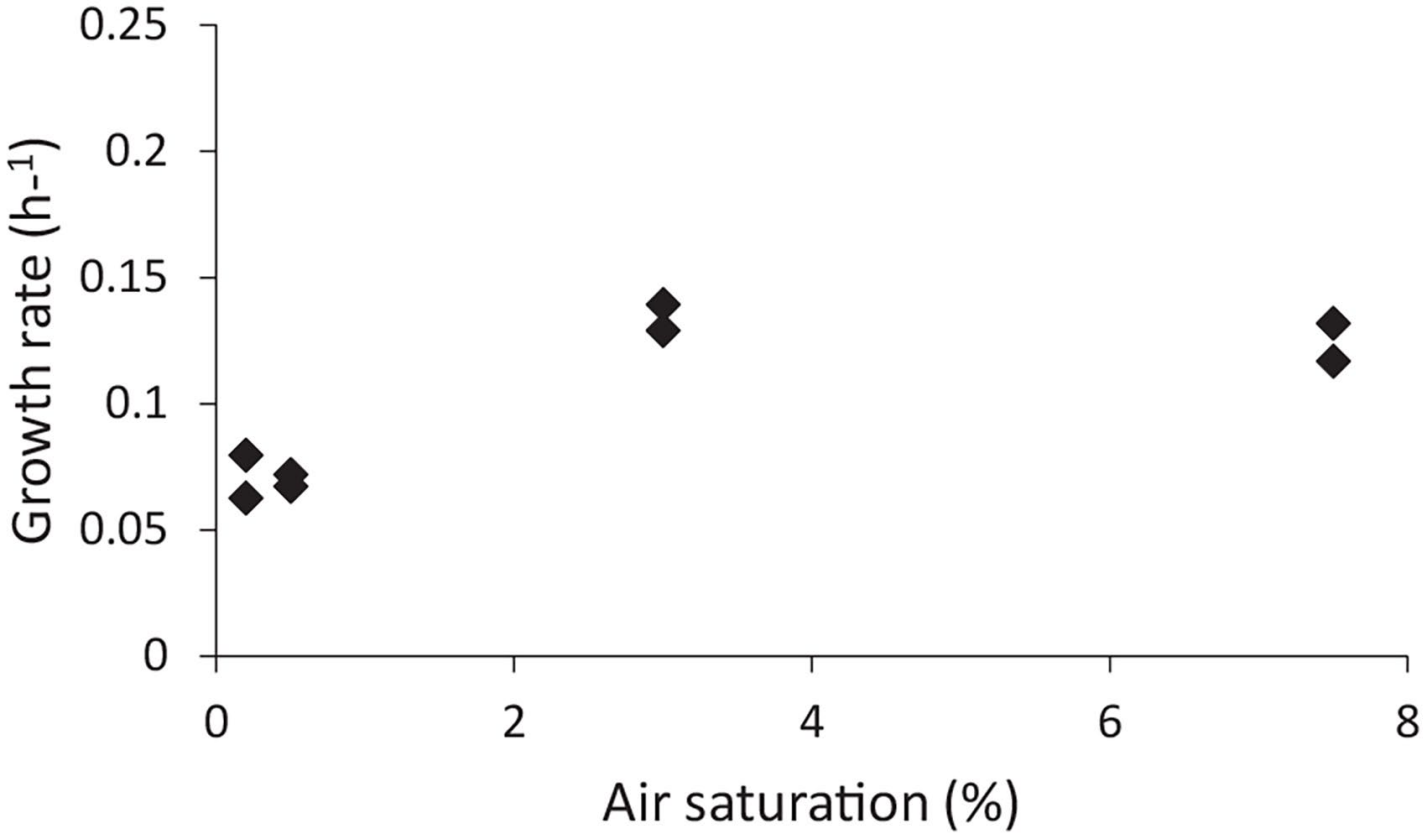
The effect of dissolved O_2_ concentration on the growth rate.

Group 1b hydrogenases are classified as oxygen sensitive, whereas group 1 h/5 hydrogenases are oxygen tolerant (Greening et al., 2014; Mohammadi et al., 2017; Schmitz et al., 2020). It was tested if the expression of the group 1b hydrogenase was affected by the dissolved O_2_ concentration and if, in turn, this would have an impact on the growth rate. The effect of O_2_ on growth rate was tested in a bioreactor, whereby the dissolved oxygen concentration was controlled. Growing “*Ca*. H. filiaventi” R50 at low dissolved O_2_ concentration (0.2% or 0.5% AS) resulted in a lower growth rate compared to the growth rate obtained at higher dissolved O_2_ concentrations (3% or 7.5% AS), whereby a maximal growth rate of 0.14 h^−1^ (doubling time 5.0 h) was obtained (Figure 7). To test whether both hydrogenases were expressed and to see if the expression levels were influenced by the dissolved O_2_ concentration, a transcriptome analysis was performed. This analysis showed that both hydrogenases (1b and 1 h/5) were constitutively expressed during growth in batch with 0.2 and 7.5% air saturation and during growth in a chemostat with O_2_ as limiting substrate (DO 0%). No increase in the expression levels of the oxygen sensitive group 1b hydrogenase was observed with decreasing O_2_ concentration.

### Kinetics of H_2_ consumption

**Figure 8:**
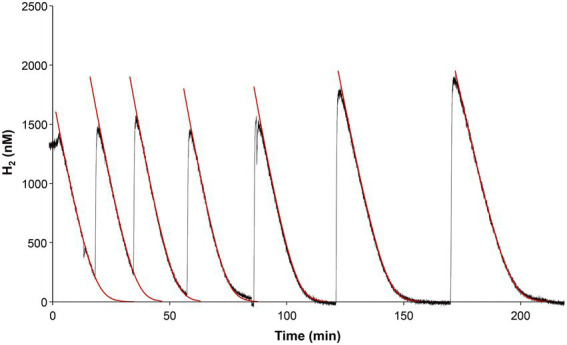
H_2_ uptake by “*Ca*. H. filiaventi” R50 in the MIMS-system. The raw data are shown by the black lines. After each addition of new substrate (hydrogen) iterative curve fitting (1 s time intervals) was performed (red lines). The substrate drop per second was calculated based on Michaelis–Menten kinetics. Each time interval (second) represents a new rate and will provide the substrate consumption over that period. The first part of each red line is virtual linear and as such represents V_(app)max_. V_(app)max_ and K_(app)s_ in the kinetic formula were adapted to obtain the best fit through the raw data. The first four rounds of H_2_ consumption were used to determine the V_(app)max_, whereas the last four rounds of H_2_ consumption were used to calculate the K_(app)s_.

“*Ca*. H. filiaventi” R50 possesses a high affinity group 1 h/5 hydrogenase, which are known for their extreme high affinity for H_2_ (Schmitz et al., 2020). The test if “*Ca*. H. filiaventi” R50 cells have a high affinity for H_2_, the kinetic parameters for H_2_ consumption were measured using Membrane-Inlet Mass Spectrometry (MIMS) with cells from a H_2_-limited chemostat (5% AS). Addition of H_2_ resulted in immediate H_2_ consumption. The V_max_ could only be determined accurately if H_2_ was not consumed completely and oxygen concentrations were 0.3–0.4% AS (Figure 8). After the biomass had been without H_2_, the V_(app)max_ was reduced, but the K_(app)s_ was not affected. Therefore, the V_(app)max_ and the K_(app)s_ could be determined after each other in the same experiment (Figure 8). The H_2_ depletion followed Michaelis–Menten kinetics and resulted in a V_(app)max_ of 953 ± 3 nM H_2_/min/mg dry weight (*n* = 4) and strain R50 has a high affinity for H_2_, since a K_(app)s_ of 353 ± 4 nM (*n* = 4) was found.

### Carbon fixation

**Figure 9:**
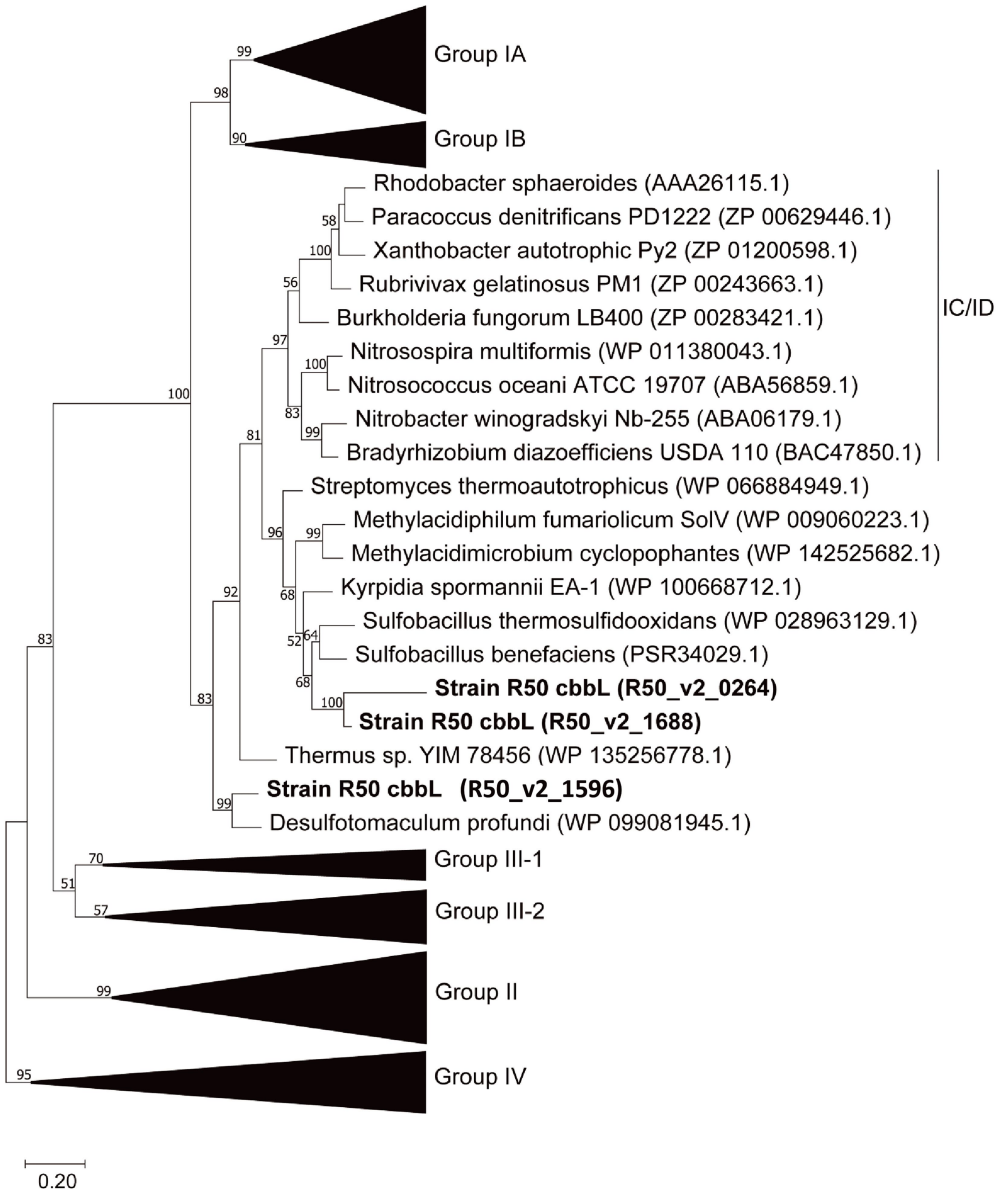
Phylogenetic analysis of the RubisCO large subunit (CbbL) using the classification as proposed by Tabita et al. (2007). The amino acid-based tree was constructed using the Maximum Likelihood method (Saitou and Nei, 1987). Bootstrap percentage values above 50% (1,000 replicates) are given at each node. Evolutionary distances were calculated using the Poisson correction method (Zuckerkandl and Pauling, 1965). The analysis was performed using MEGAX (Kumar et al., 2018). Group IV RuBisCO was used to root the tree.

“*Ca*. H. filiaventi” R50 uses the Calvin-Benson-Bassham (CBB) cycle for carbon fixation. The first enzyme of the CBB cycle is the ribulose-1,5- bisphosphate carboxylase (cbb) composed of a large chain (CbbL) and a small chain (CbbS). The genome encodes for three *cbbL* genes and two *cbbS* genes (Supplementary Table S1). Phylogenetic analysis of the genes encoding the large subunit (*cbbL*) showed that these genes cluster within the group I *cbbL* 70–84% identity, but might represent novel subgroups together with other thermophiles (Figure 9). Especially R50_v2_1596 even may belong to a different subgroup outside of Form IC/D, Transcriptomic analysis showed that only two *cbbL* genes (R50_v2_0264 and R50_v2_1688) and one *cbbS* gene (R50_v2_1687) were expressed during batch growth and growth under O_2_ limitation in a chemostat (Table 2). The expression values of R50_v2_1596 (*cbbL*) and R50_v2_1595 (*cbbS*) are low under these conditions. All other CBB cycle genes were expressed, whereby glyceraldehyde-3-phosphate dehydrogenase (*gapA*, R50_v2_2224) fructose-bisphosphate aldolase (*fba*, R50_v2_1401), ribulose-5-phosphate 3-epimerase, (rpe, R50_v2_1689), and phosphoribulokinase (*prk*, R50_v2_1690) showed high expression values (Table 3).

**Table 3:** Expression levels of the genes involved in H_2_ oxidation and CO_2_ fixation in strain R50.

Pathway	Product	Gene name	Identifier MicroScope	Expression level (RPKM)		
				Batch 0.2% AS	Batch 7.5% AS	Chemostat O_2_-lim
1b H2ase	Hydrogenase formation chaperone	*hypC*	R50_v2_1061	297 ± 106	484 ± 146	452 ± 65
	Carbamoyltransferase, hydrogen maturation protein	*hypE*	R50_v2_1062	117 ± 45	205 ± 110	265 ± 77
	Hydrogenase small subunit	*hynA*	R50_v2_1064	101 ± 61	285 ± 201	402 ± 187
	Hydrogenase large subunit-group 1b	*hynB*	R50_v2_1065	125 ± 76	328 ± 231	413 ± 188
	Cytochrome b subunit	*hynC*	R50_v2_1066	233 ± 100	475 ± 219	531 ± 228
	Hydrogenase maturation protein	*hupD*	R50_v2_1067	279 ± 114	448 ± 196	466 ± 186
	Hydrogenase accessory protein	*hypC*	R50_v2_1068	107 ± 42	214 ± 129	166 ± 72
	Hydrogenase maturation factor	*hypA*	R50_v2_1072	177 ± 63	228 ± 75	287 ± 58
	Hydrogenase maturation factor	*hypB*	R50_v2_1,073	50 ± 17	66 ± 19	84 ± 13
1 h/5 H2ase	Hydrogen maturation factor	*hypB*	R50_v2_2276	34 ± 1 3	57 ± 49	46 ± 12
	Hydrogen maturation factor	*hypA*	R50_v2_2277	0.5 ± 0.5	0.25 ± 0.43	1 ± 0
	Carbamoyltransferase, hydrogen maturation protein	*hypE*	R50_v2_2278	18 ± 9	21 ± 16	26 ± 9
	Hydrogenase maturation factor	*hypD*	R50_v2_2279	61 ± 31	81 ± 63	85 ± 25
	Hydrogenase accessory protein	*hypC*	R50_v2_2280	124 ± 87	118 ± 75	164 ± 37
	Hydrogenase large subunit - group 1 h/d	*hhyL*	R50_v2_2282	462 ± 165	605 ± 427	696 ± 148
	Hydrogenase small subunit	*hhyS*	R50_v2_2283	3,432 ± 110	386 ± 130	550 ± 69
CBB cycle	Ribulose bisphosphate carboxylase large chain	*cbbL*	R50_v2_0264	214 ± 25	314 ± 31	183 ± 24
	Ribulose bisphosphate carboxylase small chain	*cbbS*	R50_v2_1595	8 ± 7	25 ± 16	3 ± 1
	Ribulose bisphosphate carboxylase large chain	*cbbL*	R50_v2_1596	9 ± 3	35 ± 25	5 ± 3
	Ribulose bisphosphate carboxylase small chain	*cbbS*	R50_v2_1687	245 ± 53	340 ± 87	193 ± 8
	Ribulose bisphosphate carboxylase large chain	*cbbL*	R50_v2_1688	48 ± 16	58 ± 28	48 ± 9
	Phosphoglycerate kinase	*pgk*	R50_v2_2223	8 ± 1	8 ± 2	12 ± 3
	Glyceraldehyde-3-phosphate dehydrogenase	*gapA*	R50_v2_2224	117 ± 10	120 ± 45	131 ± 17
	Triose phosphate isomerase	*tpiA*	R50_v2_2222	5 ± 1	5 ± 2	9 ± 2
	Fructose-bisphosphate aldolase	*fba*	R50_v2_1401	92 ± 20	118 ± 42	127 ± 18
	Fructose-bisphosphate aldolase	*fba*	R50_v2_1599	0.25 ± 0.43	0 ± 0	0 ± 0
	Fructose 1,6-bisphosphatase class II	*glpX*	R50_v2_0617	47 ± 23	29 ± 11	32 ± 6
	Transketolase	*tktA*	R50_v2_1691	24 ± 6	29 ± 11	42 ± 10
	Triose phosphate isomerase	*rpiB*	R50_v2_0628	97 ± 24	66 ± 13	81 ± 15
	Ribulose-5-phosphate 3-epimerase	*rpe*	R50_v2_1689	239 ± 85	137 ± 24	175 ± 36
	Phosphoribulokinase	*prk*	R50_v2_1690	452 ± 84	284 ± 32	353 ± 60
Valine/leucine/isoleucine biosynthesis	Acetolactate synthase (large subunit)	*ilvB*	R50_v2_0814	30 ± 13	13 ± 4	25 ± 5
	Acetohydroxy-acid isomeroreductase (NADP-dependent)	*ilvC*	R50_v2_0811	58 ± 33	12 ± 5	54 ± 12
	Dihydroxy-acid dehydratase	*ilvD*	R50_v2_0815	35 ± 10	19 ± 7	32 ± 7
	Branched-chain-amino-acid aminotransferase	*ilvE*	R50_v2_0816	35 ± 13	23 ± 9	36 ± 7
	Branched-chain-amino-acid aminotransferase	*ilvE*	R50_v2_2305	35 ± 11	28 ± 13	38 ± 10
	Leucine dehydrogenase, NAD-dependent	*bcd*	R50_v2_1159	5 ± 1	8 ± 3	14 ± 4
	2-isopropylmalate synthase	*leuA*	R50_v2_0812	32 ± 22	11 ± 4	24 ± 8
	3-isopropylmalate dehydratase (large subunit)	*leuC*	R50_v2_1801	26 ± 7	15 ± 5	21 ± 6
	3-isopropylmalate dehydrogenase	*leuB*	R50_v2_0813	18 ± 9	6 ± 2	15 ± 3

The mRNA expression is shown as RPKM values (*n* = 3 ± standard deviation). RNA-seq was performed on a batch operated at 0.2% air saturation (AS), a batch at 7.5% air saturation and an O_2_-limited chemostat culture.

CO_2_ fixation through the CBB-cycle is the first step in the production of the excreted organic compounds. Extensive analysis with LC–MS and NMR demonstrates that these excreted compounds consist of amino acids, with Ile, Val and Ala being the most dominant. All three amino acids are synthesized from pyruvate and their production share enzymatic conversions. The genome of H. filiaventi R50 encodes the whole valine, leucine, and isoleucine biosynthesis pathway (Supplementary Table S1). The valine, leucine, and isoleucine biosynthesis pathways were expressed under all tested conditions and the expression values of this pathway are comparable under all three different conditions (Table 3).

### Metabolic potential

The substrate range that can be used by “*Ca*. H. filiaventi” R50 seems to be very narrow. Growth on sugars was not observed. Genome analysis revealed that sugar transporters are absent and the glycolysis and gluconeogenesis pathways are incomplete. Glucose-6-phosphate isomerase is missing, which catalyzes the reversible conversion of glucose-6-P into fructose-6-P. No genes were detected that encode for an enzyme that transfers fructose-6-P into fructose-1,6-P. The gene for the enzyme that catalyzes the reverse reaction, fructose-1,6-bisphosphatase could be detected (Supplementary Table S1). Transcriptome analysis showed that the expression values of the genes of the glycolysis and gluconeogenesis are low, unless these genes are shared with the CBB cycle (Table 3). The TCA cycle is complete and expressed under all tested growth conditions (Supplementary Tables S1). Genes encoding the enzymes of the glyoxylate shunt are not present in strain R50. The genes for pentose phosphate pathway enzymes were detected in strain R50 and expressed under all conditions tested (Supplementary Tables S1).

"*Ca.* Hydrogenisulfobacillus filiaventi" R50 used ammonium as nitrogen source. The genome contained three ammonium transporters of which all were expressed under the tested growth conditions. Glutamate dehydrogenase (R50_v2_0919) and glutamine synthetase (R50_v2_1125, R50_v2_2306) are used for primary ammonium assimilation. Nitrate, nitrite, urea and N_2_ could not be used as nitrogen source during growth in batch, despite the presence of genes that should support growth on these compounds. The genome contains urease genes (*ureABCDG*, R50_v2_2255, R50_v2_2256, R50_v2_2259–2,261) and nitrogenase genes (*nifCDEHKXZ*, R50_v2_0452, R50_v2_0454–0459, R50_v2_0464). Genes for partial denitrification were detected, including a nitrate/nitrite transporter (R50_v2_1496), nitrate reductase (*narGHIJK*, R50_v2_1497, R50_v2_1497–1,501), nitrite reductase (*nirK*, R50_v2_1317), and nitric oxide reductase (*norB*, R50_v2_0576; Supplementary Table S1). However, no anaerobic growth on nitrate or nitrite was observed under the tested conditions, nor was there any physiological evidence for partial denitrification.

“*Ca*. H. filiaventi” R50 could not use sulfate as sulfur source, since the sulfate assimilation pathway is absent. Instead, the genome encodes for thiosulfate reductase (prsABC, R50_v2_1049–1,051), tetrathionate hydrolase (tth, R50_v2_2562), thiosulfate dehydrogenase (R50_v2_2558, R50_v2_2559), sulfur oxygenase/reductase (sor, R50_v2_0249, R50_v2_1684), and sulfite dehydrogenase (soeAB, R50_v2_0555, R50_v2_0556, R50_v2_1810, R50_v2_1811) to support the growth on thiosulfate, tetrathionate, and sulfur. Cysteine kinase (*cysK*, R50_v2_1277, R50_v2_1328) assimilates sulfide into cysteine (Supplementary Table S1). Also, cysteine can function as sulfur source, but not as energy source. Thiosulfate, tetrathionate, and sulfide could not be used as electron donor either.

## Discussion

In this study, a novel aerobic, thermoacidophilic H_2_-oxididizing bacterium “*Ca*. Hydrogenisulfobacillus filiaventi” R50 gen. nov., sp., nov., was isolated from the geothermal soil of the Favara Grande on Pantelleria Island, Italy. This strain was isolated using a continuous culture with H_2_ as limiting substrate followed by serial dilution to extinction. “*Ca*. H. filiaventi” R50 used CO_2_ as carbon source and converted half of the fixed CO_2_ into biomass, whereas the other half was excreted as various amino acids. As a consequence of this excretion, the biomass yield on H_2_ is much lower (1.4 g DW/mol H_2_) compared to other “Knallgas” bacteria, such as *Ralstonia eutropha* (4.6 g DW/mol H_2_) or *Methylacidiphilum fumariolicum* SolV (3.4 g DW/mol H_2_), (Morinaga et al., 1978, Mohammadi et al., 2017). In all cases biomass yields were determined under oxygen-limited conditions but it should be considered that some variability may be caused by availability of H_2_ and inorganic nutrients.

“*Ca*. H. filiaventi” R50 has an optimal growth temperature of 55°C and only grows between pH 2.5 and 4.0. These extreme conditions are observed in the soil where “*Ca*. H. filiaventi” R50 was isolated from (Gagliano et al., 2014). Interestingly, despite the fact that many “Knallgas” bacteria can grow on a variety of organic substrates (Bowien and Schlegel, 1981; Bonjour and Aragno, 1984; Friedrich and Schwartz, 1993; Reiner et al., 2018; Hogendoorn et al., 2020), “*Ca*. H. filiaventi” R50 cannot grow on sugar, volatile fatty acids or alcohols under the tested conditions. These organic substrates might not be available in great access in the natural habitat of “*Ca*. H. filiaventi” R50, which could have resulted in loss of the genetic blueprint to grow on these compounds. Despite having the genes that indicate that strain R50 could use N_2_ and urea for growth, and is capable of partial denitrification, this could not be confirmed in batch cultivation experiments. Nitrogenases are O_2_ sensitive and might only be active at very low O_2_ concentration, as was observed in *Methylacidiphilum fumariolicum* SolV (Khadem et al., 2011). Denitrification is often hampered at low pH and it might require a long time before the expression of denitrification genes is started (van den Heuvel et al., 2010).

The genome “*Ca*. H. filiaventi” R50 encodes for two hydrogenases, one classified as oxygen-sensitive group 1b hydrogenase and one as oxygen tolerant, high affinity, group 1 h/5 hydrogenase (Greening et al., 2016). Despite that the 1b hydrogenase is classified as oxygen sensitive, this hydrogenase was expressed under O_2_-limited conditions, at 0.2% air saturation and 7.5% air saturation. This indicates that the expression of this hydrogenase is not controlled by oxygen concentration. The same was reported for transcription and translation of NiFe hydrogenases from *Sulfurospirillum multivorans* (Kruse et al., 2017). Furthermore, there is no physiological evidence that supports the oxygen sensitive character of this 1b hydrogenase. The growth rate of “*Ca*. H. filiaventi” R50 does not increase when the oxygen concentration is reduced. This is in contrast to *Methylacidiphilum fumariolicum* SolV that possesses besides a 1 h/5 hydrogenase, an oxygen-sensitive 1d hydrogenase. For *M. fumariolicum* SolV, the growth rate increased at lower oxygen concentration due to the increased activity of its oxygen-sensitive 1d hydrogenase (Mohammadi et al., 2017). Group 1 h/5 hydrogenases are known for their extremely high affinity for H_2_ and are held responsible for atmospheric H_2_ uptake. Atmospheric hydrogen gas (H_2_) concentrations are only 0.53 ppmv (Novelli et al., 1999), but despite this low concentration, soil microorganisms can consume these trace amounts of H_2_ (Schuler and Conrad, 1990; Conrad, 1996). Tropospheric H_2_ concentrations can be scavenged by different soil microorganisms with a 1 h/5 hydrogenases, which gives them this extremely high affinity for H_2_. “*Ca*. H. filiaventi” R50 has a high affinity for H_2_, with a K_s_ of 353 nM. Such a high affinity is observed in different soil microorganisms, including the Acidobacteriota *Streptomycetes* (Constant et al., 2010) and *Acidobacterium ailaaui* (Myers and King, 2016), the Actinomycetota *Mycobacterium smegmatis* (Greening et al., 2014), the Chloroflexi *Thermomicrobium roseum* and *Thermogemmatispora* sp. T81 (Islam et al., 2019), and the Verrucomicrobiota *Methylacidiphilum fumariolicum* SolV (Schmitz et al., 2020). All of these microorganisms possess a group 1 h/5 hydrogenase. Geothermal environments are usually exposed to elevated H_2_ concentrations, but the emitted H_2_ concentration might fluctuate over time. Having a high affinity hydrogenase might not only sustain growth on H_2_, but also be involved in persistence when the H_2_ concentrations are too low to grow on (Greening et al., 2014, 2016; Schmitz et al., 2020).

“*Ca*. H. filiaventi” R50 uses the Calvin-Benson-Bassham (CBB) cycle for CO_2_ fixation with the key enzymes ribuolose-1,5-bisphosphate carboxylase (RubisCO) and phosphoribulokinase. RubisCO incorporates CO_2_ into ribulose-1,5-bisphosphate to produce two 3-phosphoglycerate molecules. Eventually, the CBB cycle yields one glyceraldehyde-3-phosphate at the expense of nine ATP equivalents and six NAD(P)H (Wildman, 2002; Berg, 2011). So far, four different forms of RubisCO have been described, whereby form I and form II play a role in autotrophic carbon fixation (Tabita et al., 2007). Form I is comprised a large and a small subunit, whereas form II consists of multimers of the large subunit. Furthermore, the role of these two RuBisCO forms differs. Form I is mainly involved in the carbon fixation for biomass production, whereas from II is involved in balancing the redox potential, whereby CO_2_ is used as electron sink (Tabita, 1999; Dubbs and Tabita, 2004). “*Ca*. H. filiaventi” R50 encodes three different RubisCO large subunits and two different small subunits. All of these cluster closely to the group 1C/D RubisCO and are likely to be involved in CO_2_ fixation for the production of larger molecules. Two of the three RubisCO large genes are expressed under the tested conditions. The expression of the different RubisCO genes might be dependent on CO_2_ levels, as in the autotrophic hydrogen oxidizer *Hydrogenovibrio marinus.* This bacterium possess two copies of form I RubisCO and one copy of form II RubisCO. Form II is expressed at high (>15%) CO_2_ concentrations, one of the form I RubisCO genes was expressed below 0.15% CO_2_ and the other form I RubisCO was expressed in the intermediate CO_2_ concentrations. Interestingly, at atmospheric CO_2_ concentration, all three RubisCO genes are expressed in *Hv. marinus* (Yoshizawa et al., 2004).

Fixation of CO_2_ by RubisCO is the first step in the production and excretion of the unknown organic compounds. Extensive chemical analysis revealed that these organic compounds are amino acids and Hse-lactone. It is remarkable, that there are so far no indications of feedback regulation found, since the production of these organic compounds from CO_2_ is an energy intensive process. Transcriptome analysis showed expression of the valine/leucine/isoleucine pathways during batch growth and under O_2_-limited conditions.

The ecological reason for the excretion of high amounts of organic compounds remains uncertain. It could be a compound that is involved in signaling, stimulates symbioses, or has antimicrobial properties (Berdy, 2005). The production of these molecules can be the results of electron balancing. In other words, the generated electrons during the oxidation of H_2_ cannot all be transferred to O_2_, hence these organic molecules are excreted to get rid of the electrons. Remarkably, we did not observe the metabolic potential for carbon (glycogen, PHB) and/or energy (polyphosphate) storage. Nevertheless, it is very likely that the growth conditions in the laboratory, are not the growth conditions that this strain faces in their natural habitat. In fact, it is likely that the strain is often in dormant state, rather than growing at high growth rate. It might be that under more natural growth conditions, these amounts of organic compounds are not excreted.

Using the CBB-cycle for CO_2_ fixation, high input of ATP and reducing equivalents are needed. These come from the oxidation of the electron donor, in this case H_2_. To use an autotrophic “Knallgas” bacterium for the economically feasible production of chemicals, the production of H_2_ must be inexpensive. Furthermore, sustainable resources, such as solar and wind power cannot only be used for energy generation, but also for the production of H_2_. In addition, CO_2_ levels must be high enough to reach the highest CO_2_ fixation rates. Special bioreactors must be developed in order to supply sufficient substrates (H_2_, CO_2_ and O_2_) to the microbial culture, since these gasses dissolve poorly in water. Nevertheless, using this H_2_ for the production of chemicals, while fixing CO_2_, could be a sustainable alternative to the production of chemicals from oil or natural gas. However, more research will be needed to evaluate if our autotrophic “*Ca*. Hydrogenisulfobacillus filiaventi” strain R50 could provide a new sustainable alternative.

## Data availability statement

The datasets presented in this study can be found in online repositories. The names of the repository/repositories and accession number(s) can be found in the article/Supplementary material.

## Author contributions

CH, AP, MJ, and HC designed the projects and experiments. AP, CH, and HC sampled the geothermal soils. CH and AP performed the enrichment and isolation experiments. CH and AP conducted the physiology experiments, including MIMS. RM performed the electron microscopic analyzes. RG, PW, RM, PG, and RJ performed the NMR and untargeted metabolomics experiments. GC and TA sequenced the genome and transcriptome, reconstructed the genome, and analyzed the gene expression. CH, AP, and HC carried out the data analysis. CH, MJ, and HC wrote the manuscript. All authors contributed to revision of the manuscript, and read and approved the submitted version.

## Funding

CH and HC were supported by the European Research Council (ERC Advanced Grant project VOLCANO 669371), MJ was supported by the European Research Council (ERC Advanced Grant project Eco_MoM 339880) and The Soehngen Institute of Anaerobic Microbiology (SIAM 024002002).

## Conflict of interest

The authors declare that the research was conducted in the absence of any commercial or financial relationships that could be construed as a potential conflict of interest.

## Publisher’s note

All claims expressed in this article are solely those of the authors and do not necessarily represent those of their affiliated organizations, or those of the publisher, the editors and the reviewers. Any product that may be evaluated in this article, or claim that may be made by its manufacturer, is not guaranteed or endorsed by the publisher.
