# Supplementary material for: “Candidatus Hydrogenisulfobacillus filiaventi” strain R50 gen. nov. sp. nov., a highly efficient producer of extracellular organic compounds from H2 and CO2
Source: Front Microbiol. 2023 Mar 24;14:1151097. doi: 10.3389/fmicb.2023.1151097 (PMC10080006; doi:10.3389/fmicb.2023.1151097)
Supplement: Supplementary file 2 [file Data_Sheet_1.PDF]

Supplementary Material

Hydrogenobacillus filiaventi strain R50 gen. nov. sp. nov., a highly efficient producer of extracellular organic compounds from H_2 an CO_2

Carmen Hogendoorn[1], Arjan Pol[1], Rob de Graaf[1], Paul B. White[2], Rob Mesman[1], Peter M. van Galen[3], Theo A. van Alen[1], Geert Cremers[1], Robert S. Jansen[1], Mike S.M. Jetten[1] & Huub J.M. Op den Camp[1*]

[1] Department of Microbiology, RIBES; [2] Department of Synthetic Organic Chemistry, IMM; [3] Department of Systems Chemistry, IMM, Faculty of Science, Radboud University, Heyendaalseweg 135, 6525 AJ Nijmegen, Netherlands

*** Correspondence: h.opdencamp@science.ru.nl**

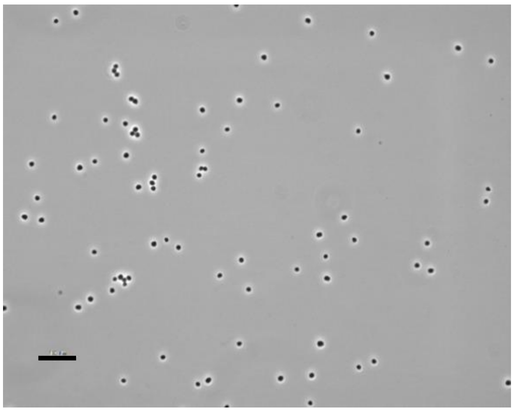

Supplementary Figure S1: Microscopy image of the *Acidianus* sp. enrichment culture. The bar resembles 5 µm.

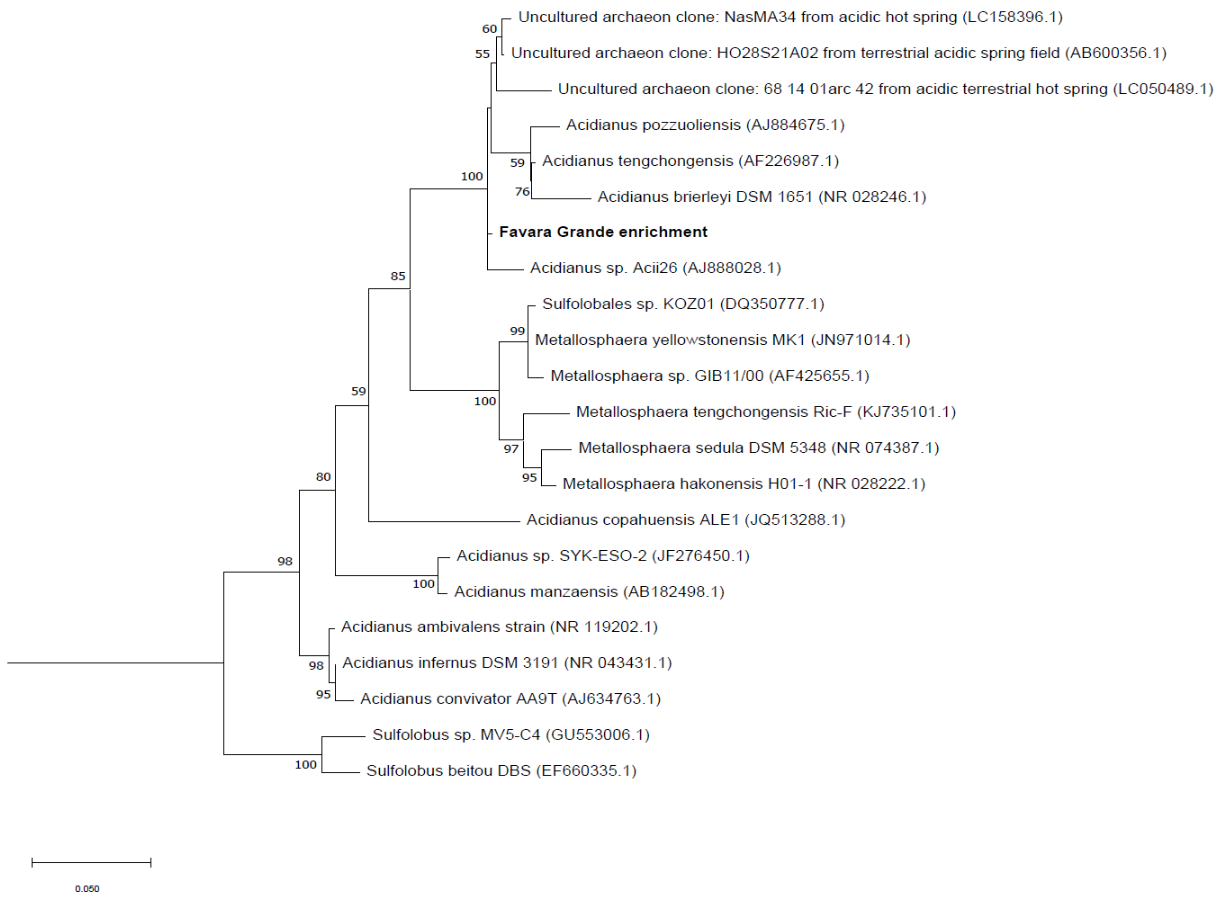

Supplementary Figure S2: Phylogenetic analysis of the 16S rRNA gene of the enriched *Acidianus* sp. and its close relatives. The tree was constructed using the Maximum Likelihood method and Tamura-Nei model (Tumaru 1993). Bootstrap percentage values above 50% (1000 replicates) are given at each node. The analysis involved 23 nucleotide sequences and was performed using MEGAX (Kumar 201). The 16S rRNA sequence of the [Fe]-hydrogenase *Methanocella conradii* (NR_118245.1) was used to root the tree, but the branch was removed for clarification.

```
>Favare Grande enrichment, partial 16S rRNA
TGGGAGTCGTACGCTCTCGGTAAGAGGGCGTGGCGGACGGCTGAGTAACACGTGGTCAACCTAACCTCGGGACTTGGATA
ACTCCGGGAAACTGGAGCTAATCCAAGATAGGCAAAGGAATCTGGAACGATTCTTTGCCCAAAAGCCTCTAGGCTAATAC
TGTCTASAGGTGCCCGAGGATGGGACTGCGGCCCATCAGGCTGNNNNNNNNNNNNNNNNNNNNNNNNNNNNNNNNGGGTA
GGGGCCGTGGGAGCGGGAGCCCCCAGTTGGGCACTGAGACAATGGCCCAGGCCCTACGGGGCGCACCAGGCGCGAAACGT
CCCCAATGCGGGAAACCGTGAGGGCGCTATCCCCAGTGCCTCCGATAGGAGGCTTTTCCCCACTTTAGAACGGTGGGGGA
ATAAGCGGGGGGCAAGGCTGGTGTCAGCCGCCGCGGTAATACCAGCCCCGCGAGTGATCTGGACGTTTATTGGGCTTGAA
GCGCCCGTAGCCGGCCCATAAAGTCACTGTTTAAAGACCCGGGCTCAACCCGGGAAAGGGCAGTGATACTTATGGGCTAG
GGGGCGGGAAAGGTCGGAGGTACTCCCGGAGTAGGGGCGAAATCCGTAGATCCCGGGAGGACCACCAGTGGCGAAAGCGT
CCGGCTAGAACGCGCCCGACGGTGAGGGGCGAAAGCCGGGGCAGCAAAAGGGATTAGATACCCCTGTAGTCCCGGCTGTA
AACGATGCAGGCTAGGTGTCGCGTGGGTCTAGAGCCCGCGCGGTGCCGCAGGGAAACTGGTAAGCCCGCCGCC
```

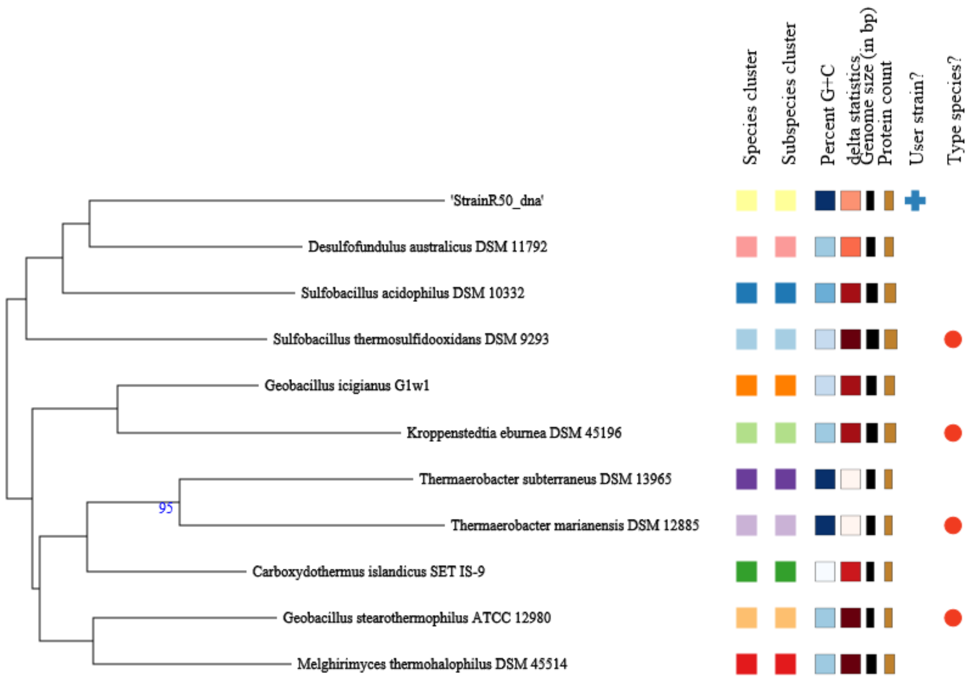

Supplementary Figure S3. Tree inferred with FastME 2.1.6.1 from TYGS-GBDP distances calculated from genome sequences. The branch lengths are scaled in terms of GBDP distance formula d5. The numbers above branches are GBDP pseudo-bootstrap support values > 60 % from 100 replications, with an average branch support of 40.6 %. The tree was rooted at the midpoint.

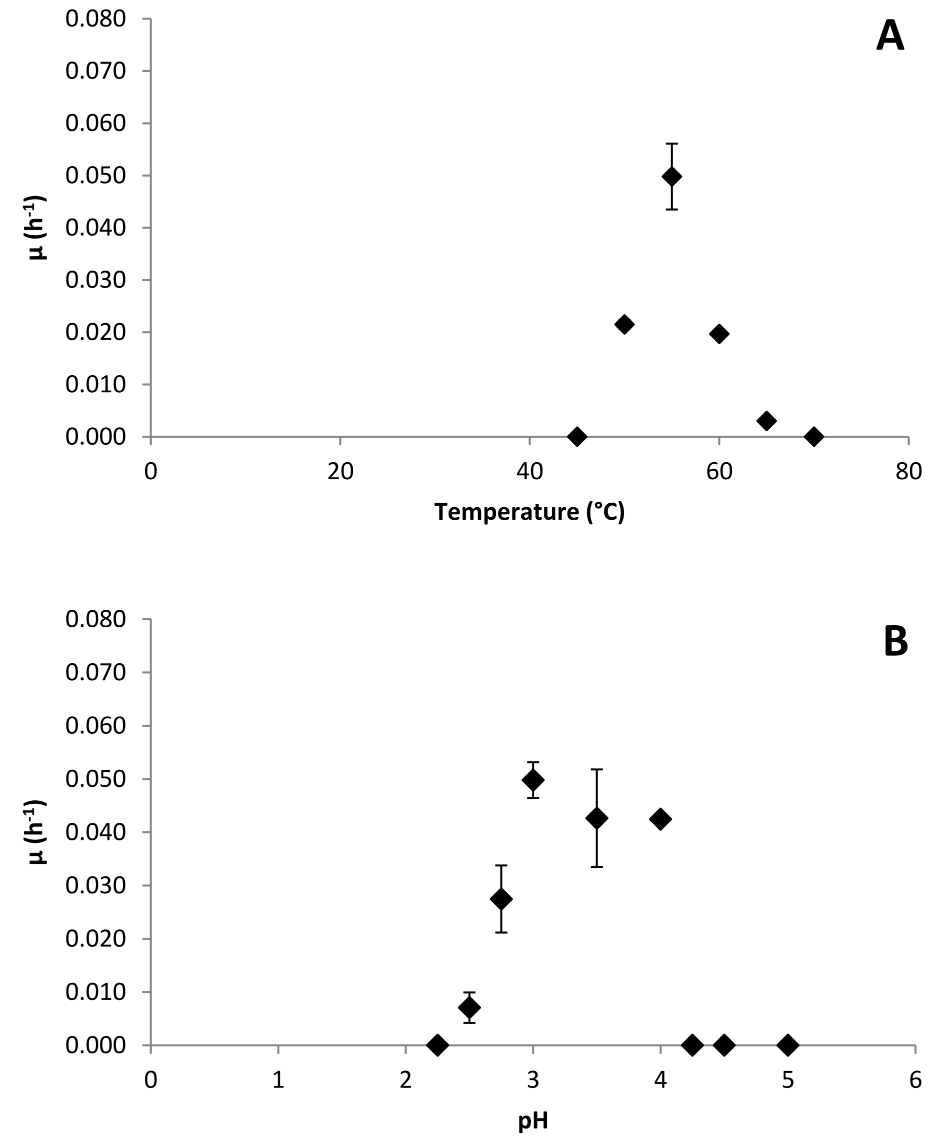

Supplementary Figure S3: A. Temperature and **B.** pH optimum for growth of strain R50. Values shown are the average of three experiments. The bar shows the standard deviation. If no bar is present, the standard deviation is smaller than the data point.
